# Narrative Review of Primary Preventive Interventions against Water-Borne Diseases: Scientific Evidence of Health-EDRM in Contexts with Inadequate Safe Drinking Water

**DOI:** 10.3390/ijerph182312268

**Published:** 2021-11-23

**Authors:** Emily Ying Yang Chan, Kimberley Hor Yee Tong, Caroline Dubois, Kiara Mc Donnell, Jean H. Kim, Kevin Kei Ching Hung, Kin On Kwok

**Affiliations:** 1 Collaborating Centre for Oxford University and CUHK for Disaster and Medical Humanitarian Response, Hong Kong, China; kevin.hung@cuhk.edu.hk; 2 Nuffield Department of Medicine, University of Oxford, Oxford OX3 7BN, UK; 3 JC School of Public Health and Primary Care, Faculty of Medicine, The Chinese University of Hong Kong, Hong Kong, China; 1155157029@link.cuhk.edu.hk (K.H.Y.T.); caroline.dubois@gxfoundation.hk (C.D.); JHKim@cuhk.edu.hk (J.H.K.); kkokwok@cuhk.edu.hk (K.O.K.); 4 GX Foundation, Hong Kong, China; kiaramcd@yahoo.com; 5 Accident & Emergency Medicine Academic Unit, The Chinese University of Hong Kong, Prince of Wales Hospital, Hong Kong, China; 6 Stanley Ho Centre for Emerging Infectious Diseases, The Chinese University of Hong Kong, Hong Kong, China; 7 Shenzhen Research Institute, The Chinese University of Hong Kong, Hong Kong, China; 8 Hong Kong Institute of Asia-Pacific Studies, The Chinese University of Hong Kong, Hong Kong, China

**Keywords:** biological hazard, primary prevention, health-EDRM, water-borne disease, diarrheal disease, safe drinking water

## Abstract

Waterborne diseases account for 1.5 million deaths a year globally, particularly affecting children in low-income households in subtropical areas. It is one of the most enduring and economically devastating biological hazards in our society today. The World Health Organization Health Emergency and Disaster Risk Management (health-EDRM) Framework highlights the importance of primary prevention against biological hazards across all levels of society. The framework encourages multi-sectoral coordination and lessons sharing for community risk resilience. A narrative review, conducted in March 2021, identified 88 English-language articles published between January 2000 and March 2021 examining water, sanitation, and hygiene primary prevention interventions against waterborne diseases in resource-poor settings. The literature identified eight main interventions implemented at personal, household and community levels. The strength of evidence, the enabling factors, barriers, co-benefits, and alternative measures were reviewed for each intervention. There is an array of evidence available across each intervention, with strong evidence supporting the effectiveness of water treatment and safe household water storage. Studies show that at personal and household levels, interventions are effective when applied together. Furthermore, water and waste management will have a compounding impact on vector-borne diseases. Mitigation against waterborne diseases require coordinated, multi-sectoral governance, such as building sanitation infrastructure and streamlined waste management. The review showed research gaps relating to evidence-based alternative interventions for resource-poor settings and showed discrepancies in definitions of various interventions amongst research institutions, creating challenges in the direct comparison of results across studies.

## 1. Introduction

Water-borne diseases (WBDs) are infectious diseases, such as cholera, shigella, typhoid, hepatitis A and E, and poliomyelitis, that are transmitted to humans through contaminated water [1]. These infections are caused by a number of bacterial, viral, and parasitic organisms where there is inadequate sanitation, hygiene, and safe water for drinking, cooking and cleaning [2]. There is a high prevalence of WBDs in low- and middle- income countries in tropical and subtropical regions. The major etiological agents for WBDs in such contexts are Rotavirus and *Escherichia coli*. Bacteria *Shigella* and parasite *Cryptosporidium* are also major agents globally [1]. A list of pathogens transmitted through water can be found in Appendix A. According to the World Health Organization (WHO), WBDs account for an estimated 3.6% of the total disability-adjusted life year global burden of disease and are the leading causes of human morbidity and mortality worldwide, causing approximately 1.5 million deaths annually [1]. Furthermore, diarrheal disease is the second leading cause of death in children under five years old [2]. It is estimated that children under three years old in low-income countries experience an average of three episodes of diarrhea annually, which can in turn, lead to malnutrition, severe dehydration and increased risk of developing deficiency disorders [3].

In many developing regions, WBDs are associated with physical water scarcity, defined as the lack of available water resources as well as economical water scarcity, defined as the lack of investment in water infrastructure for available water use [4,5,6]. It is estimated that 56% of the world’s population have unsafe sources of water, contaminated by sewage, septic tanks, latrines, or other sources [2]. In areas of water scarcity, or unsafe sanitation, populations are prone to poor hygiene practices. Specifically, there are four main transmission routes for WBDs: (1) water-borne, exposure to pathogen through ingestion of contaminated water; (2) water-washed, exposure to pathogens through a person-to-person or fecal-oral route due to poor personal hygiene; (3) water-based, exposure to pathogen through skin contact with contaminated water that has passed through an aquatic animal; and (4) water-related, insect vectors that breed near the water [7]. Worldwide, 150 million people still rely on surface water sources (i.e., lake water, ponds and springs) that possess high risk of contamination [8,9]. The lack of access to water, sanitation and hygiene (WASH) in these communities is one of the world’s most urgent public health issues, with 2.2 billion people lacking safely managed drinking water and 4.2 billion people lacking safely managed sanitation in 2015-2018 [1,9].

Socioeconomic factors can determine an individual’s access to and use of clean water, as those with lower income and educational level may be unaware to the consequences of using unsafe water and inadequate sanitation practices or infrastructure or have access to the resources necessary for improvement [5]. Other factors could further exacerbate the disease burden of WBDs in rural communities such as lack of WASH policies; poor maintenance of sanitation facilities; environmental discharges of untreated waste; and water scarcity associated with climate change [1,9]. Furthermore, WBDs can cause economic burdens and be a barrier to the socioeconomic development of communities. Loss of household income can result from cost of care and treatment, or loss of economic productivity due to sickness. The actual economic burden of WBDs is difficult to estimate due to lack of health professional capacity, under-reporting of illness in the case of asymptomatic or self-limiting infections, and non-binary diagnostic parameters [10]. However, a study conducted by the WHO Regional Office for Africa in 2005 estimated that the total economic loss due to cholera could be up to 156 million USD in the WHO African region that encompasses 47 member states [7].

The WHO Health Emergency and Disaster Risk Management (health-EDRM) Framework [11], developed in line with the Sendai Framework for Disaster Risk Reduction 2015-2030 [12], refers to the structured analysis and management of health risks brought upon by emergencies and disasters. These hazardous events can include biological hazards, such as WBDs [11,12]. The health-EDRM focuses on disease prevention through hazard and vulnerability reduction, preparedness, and response and recovery interventions, emphasizing community involvement in mitigating the burden of hazardous events. Under the health-EDRM framework, hazard preventive interventions can be implemented at three levels: primary, secondary, or tertiary prevention levels [11]. Primary prevention aims to reduce health risks and the onset of disease through health promotion, education, and awareness; secondary prevention aims to stop disease progression by screening and identifying infected individuals, while tertiary prevention focuses on treatment of disease [13]. Primary prevention, and interruption to reduce transmission, is the most cost-effective method in reducing the burden of infectious disease per capita in populations with poor access to healthcare [13,14]. Effective bottom-up approaches from an empowered community, along with top-down governance and policy, allow successful implementation of primary prevention and behavioral modification throughout the disaster management cycle: prevention, mitigation, preparedness, response and recovery [11,12,13]. Interventions that aim to improve access to WASH are main bottom-up approaches for reducing risks of WBD in endemic rural areas [15].

The United Nations Sustainable Development Goals 2015-2030 (SDG) aims to eradicate poverty and achieve a more sustainable future for all [16]. The alleviation of the burden of WBDs globally will have a cross-cutting impact on several SDGs [16]. This review examines the available published literature on primary preventive interventions against WBDs, the strength of evidence behind these interventions, and the feasibility or barriers of successfully applying health-EDRM approaches for WBD prevention in contexts with inadequate safe drinking water, or resource-poor settings.

## 2. Materials and Methods

A literature search on studies with interventions designed to reduce transmission of WBD was conducted.

### 2.1. Search Strategy

PubMed, Science Direct, Web of Science, Medline, and Scopus databases were searched in March 2021 using the MeSH key words: water, sanitation, hygiene, WASH, waterborne disease, intervention, prevention, primary prevention, measures, health-EDRM, unclean water, inadequate safe drinking water, population and community Boolean operators then combined the key words by similarity of definition into a search term: ((water AND sanitation AND hygiene) OR WASH) AND (waterborne disease) AND (intervention OR prevention OR primary prevention OR measures OR health-EDRM) AND (unclean water OR inadequate safe drinking).

### 2.2. Inclusion and Exclusion Criteria

The search was limited to human studies in international peer-reviewed journals, online reports and electronic books published in English. The search included any studies relating to any WBDs, with no distinction between causative agent or symptoms. Eligible studies were retrieved, and their bibliographies were checked for further relevant publications. To obtain the most relevant literature for this review, the titles and abstracts were screened against the inclusion and exclusion criteria.

Inclusion criteria:

1. English-language based article.
2. Published between 1 January 2000 and 24 March 2021.
3. Effectiveness of primary prevention methods against waterborne diseases mentioned in the abstract.

Exclusion criteria:

1. Abstracts that did not mention primary prevention methods against WBD.
2. Papers studying only foodborne and/or airborne diseases.
3. Papers studying secondary and/or tertiary level prevention.

Full texts of potential papers were assessed and excluded if the effectiveness of the primary prevention intervention was not reported. Through a snowballing method, further texts were identified through the references of the identified publications that fit into the inclusion criteria.

The identified papers were then categorized according to the Oxford Centre for Evidence-Based Medicine (OCEBM) 2009 Levels of Evidence (Table 1) which determines the strength of evidence of a piece of research according to its study design and methodology [17]. The papers obtained from each database were collected and consolidated, and duplicates were removed.

## 3. Results

**Figure 1:**
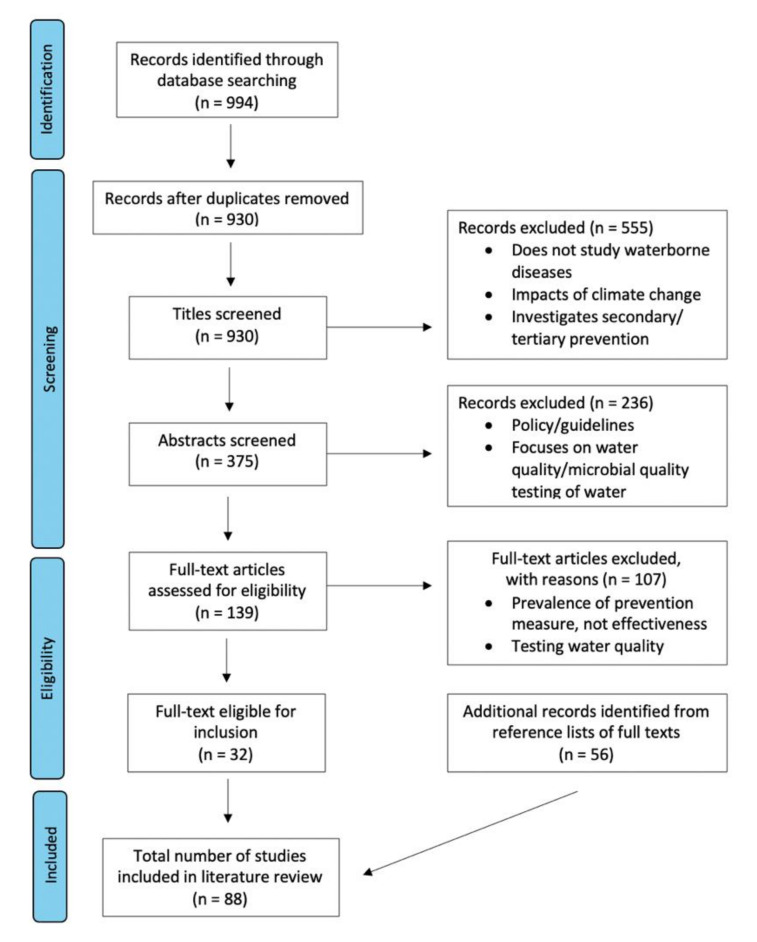
Flowchart showing the search results and exclusion process, according to databases searched, duplicates removed, publications screened, and the final number of studies included in this literature review.

The process of identifying relevant publications is outlined in Figure 1. The initial database search identified 994 search records, of which 64 were removed due to duplication. This was refined to 140 records following the screening of titles and abstracts, after which the full-texts were read and assessed for inclusion. From these results, 32 full texts were included, in addition to 56 identified through the snowballing method. The total number of studies included in this review are 88 [18,19,20,21,22,23,24,25,26,27,28,29,30,31,32,33,34,35,36,37,38,39,40,41,42,43,44,45,46,47,48,49,50,51,52,53,54,55,56,57,58,59,60,61,62,63,64,65,66,67,68,69,70,71,72,73,74,75,76,77,78,79,80,81,82,83,84,85,86,87,88,89,90,91,92,93,94,95,96,97,98,99,100,101,102,103,104,105].

### 3.1. Strength of Evidence of Identified Studies

Each of the 88 identified studies were assessed in strength of evidence of their studies, according to the OCEBM Levels of Evidence (Table S1) [17].

The included studies were categorized according to the type of intervention studied, which resulted in a group of eight common bottom-up, non-pharmaceutical, primary preventive interventions, based on the health-EDRM framework. These were: two “personal” protective practices (regular handwashing, intake of prophylactic supplements), four “household” practices (household water treatment, household water storage, maintain household cleanliness, household waste management) and two “community” practices (build community infrastructure, conduct community education) were identified. 13% of the studied literature was associated with personal practices, 65% with household practices and 22% with community practices. The review of evidence was disaggregated according to the eight preventive interventions, and categorized according to OCEBM Levels of Evidence [17], which can be found in Table 2.

The comparison of the strength of evidence of the reviewed literature (Table 2) showed that the largest proportion (35%) of identified publications fell into Level 1B classification, which includes randomized controlled trials with narrow confidence interval and the majority of these studies investigated the effects of water treatment for WBD prevention. Level 4 studies, including cross-sectional mixed-method studies and case series studies, accounted for 17% of the identified publications, which mainly evaluated the possible association between perceptions, WBD prevalence and preventive interventions in targeted populations with interviews, questionnaires and surveys. Among the 88 studies, no systematic review of case-control studies and only one systematic review of cohort studies was identified. Level 3B studies, including case-control studies, only accounted for 3% of the identified publications. There was more literature on preventive interventions at household levels (65%) with a significantly stronger study design, compared to interventions at community (22%) and personal levels (13%). Regarding individual primary preventive interventions, high-strength evidence is most available concerning the practice of water treatment, and lacking at different levels in practices of household waste management (6%) and household cleanliness (7%), with only one study available for chemoprophylaxis (0.6%).

### 3.2. Overview of Studies Included for Analysis

Table 3, Table 4, Table 5 and Table 6 summarize each of the 8 primary preventive interventions against WBDs at personal, household and community levels. Without distinction by causative agent, disease symptomology, or therapy, the tables are a compilation and comparison of each preventive methods, according to their potential health risk, desired behavioral changes, potential health co-benefits, enabling and limiting factors and strength of evidence available in published literature. The tables also identify suggested alternative measures for each intervention, which are variations of the action that have the intention of achieving a similar result, but may be implemented differently, for example, if the materials or resources required to undertake the intervention are not available or accessible.

The majority of the reviewed studies demonstrated positive relationship between primary preventive interventions on diarrhea incidence and disease transmission by addressing WBD associated health risks, however, there is a lack of assessed literature that quantifies the extent of the efficacy of such interventions on disease reduction. In the case of water treatment, many studies conferred a well-established link between less contaminated household drinking water and reduction in diarrhea risk, but not the effectiveness of WBD reduction and associated health outcomes, such as mortality, within the community [29,41,51,55,59,64,70,71,83,85,100,101].

## 4. Discussion

This narrative review examined evidence of eight primary preventive interventions against WBDs. The interventions share certain enabling and limiting factors that affect the success of proposed preventative interventions when applied to the health-EDRM framework: resources accessibility and affordability, accommodating community health facilities, correct understanding of WBD associated health risks, sustainable behavioral change, cultural relevance, and cross-sector collaboration with top-down contribution from policy makers. By contrast, socioeconomic barriers, geographical location and cultural incompetence were noted as key limiting factors.

### 4.1. Top-Down, Capacity Building, Cultural Relevance and Post-Intervention Monitoring

Many of the primary preventive interventions examined in this review were complex interventions that relied upon a combination of enabling factors to reduce WBD. For instance, a large proportion of interventions required access to material resources, ranging from simple soap to materials for constructing facilities. However, in very low-resource settings, contributions from authorities and policy-makers are also essential in order to provide these material resources. For instance, in order to ensure sustainable delivery of safe water supply and waste management systems in low income areas, multi-sectoral collaboration and coordination from local and national-level authorities is necessary. Furthermore, in order to successfully implement behavioral interventions such as the appropriate use of prophylactic supplements, government support and capacity within health system is often required. Policy makers should, therefore, re-prioritize the delivery of sustainable water and sanitation services as the importance of safe water access to reduction in WBD incidence has been reinforced in this review.

This review noted that primary interventions for reducing WBDs also often require addressing pervasive misconceptions, attitudes and social norms. For instance, WASH- education campaigns were successful in teaching participants to associate contaminated water and poor hygiene with diarrhea-related illnesses [26,28,50,77,80,99,105]. These campaigns were successful in increasing positive change in disease prevention behaviors at an individual level, as well as improvements in the hygiene practice of pupils in health education campaigns [35]. Addressing misconceptions (the perception that boiling is sufficient in killing all waterborne microbes [29]), cultural traditions (painting of mud floors with animal dung [47]) and religious beliefs (WBD outbreak as a result of ancestral curse and witchcraft [21]), allows individuals to develop understanding of the rationales behind the preventative interventions. Education and the transfer of knowledge should be delivered in a culturally-sensitive manner, whilst accounting for language needs and health literacy of the target population to guarantee accurate uptake of information [32,102]. The implementation of other primary prevention initiatives should therefore follow the health-EDRM framework with emphasis on capacity building and cultural relevance to prompt long-term positive behavioral changes [11], allowing the evaluation of the real-life impacts and feasibility of interventions. We noted in addition to cultural relevance, intervention adherence requires contextual relevance (improved buckets for water collection were more popular amongst refugee camp inhabitants despite lower effectiveness in water quality protection compared to proper chlorination, as improved features, such as small handle and lid, were more appreciated within the culture [43]). However, this review noted that in some cases, the WBD interventions lacked long-term impacts such as improvements in child health (no difference in prevalence of child diarrhea in post-intervention follow-up [76]), and improvements in hygiene practices (no difference in self-reported handwashing behavior [76], lack of adoption of water treatment into regular household routines despite distribution of filters and soap [21]). These findings may indicate decreasing compliance with interventions with time and the necessity of post-intervention small-scale monitoring to ensure sustainable positive behaviors. Hence, continued behavioral monitoring, such as regular inspection of chlorine levels in house-hold stored water, may be necessary to improve baseline water quality levels and maintain household capacity building.

### 4.2. Long-Term Sustainability and Long-Term Co-Benefits

Many It is important to note that the effect and impacts of preventive interventions are cross-cutting. The uptake of one intervention should not impede the practice of another, and despite the mixed evidence regarding the cost-effectiveness of multi-intervention programs compared to single intervention [19,28,30,34,35,39,45,46,47,56,58,59,77,87,95,99,104,105], different interventions could be promoted in rural communities to maximize the potential positive health impacts from improved water, sanitation and hygiene behavior. For instance, the construction of community infrastructures, such as filtration system that delivers clean water to storage tanks or directly to homes [54,95], and sewer system that allows safe waste disposal [67], did not only improve access to safe water but also allowed more effective uptake of certain personal and household interventions that rely on adequate baseline water quality in the community. Despite the higher costs in constructing community infrastructure, it has been shown to influence positive behavioral changes within a community (increase in the number of households with hygiene enabling facilities and proper use and maintenance of toilets and sewers [27,67]). This could reduce future expenditures on the prevention of disease outbreak or medical costs for individuals and households. Additionally, lowered medical expenses from reduced incidence of diarrheal illness can allow for greater ability to purchase resources, such as firewood and purifiers, to maintain water quality [26,48,62]. Sustainable and continuous implementation is required for all interventions to ensure maximum efficacy, and alternatives to such behavior should also be explored. Certain interventions, for example, waste management and handwashing, also exert co-benefit in reducing risks from other biological hazards under the health-EDRM framework, such as food-borne, vector-borne and droplet-borne diseases [111,112,113].

### 4.3. Research Gaps Identified in Current Published Literature

This review has identified six major research gaps in the literature relating to health-EDRM primary preventative interventions for WBDs.

First, current studies focus on reducing exposure to hazards, such as contaminated water. A total of 73% of the studies in this review proposed interventions, such as improved water treatment, water storage and waste disposal in household and community settings. There is little evaluation on the efficacy of managing other causal factors of in WBD. Future studies can examine interventions that target hazard preparedness and risk-reduction within exposed populations.

Second, research outcomes are skewed towards reduction in diarrhea incidence, with lack of evidence on the reduction of other WBD-associated symptoms, such as vomiting and stomach cramps [26,35,38]. Diarrhea is a leading cause of mortality and morbidity, especially in children under five years of age, however, it is not the sole indicator of WBD. Nor are WBDs the only cause of diarrhea, as symptoms can be associated with infectious diseases that transmit through other mechanisms, such as HIV and Ebola [114,115]. The observed reduction in incidence of solely diarrhea from an intervention does not necessarily represent the true risk reduction as related to WBDs. The impact of the intervention on WBD prevention is at risk of being overestimated if other diseases are present or underestimated if other symptoms are not considered. Future studies that evaluate the efficacy of primary prevention interventions should consider evaluating non-diarrheal symptoms such as vomiting and stomach cramps along with diarrhea, to strengthen the accuracy and validity of such methods as WBD preventative behavior, particularly in vulnerable or resource-poor communities.

Third, there is limited research on alternatives of preventive interventions for implementation in resource-poor or material-scarce settings. For example, the beneficial effect of handwashing with soap is consistent across various studies, but there is little evidence to support the use of alternatives, such as ash in communities where soap is not available [2,116]. The efficacy of such alternatives has been demonstrated in averting the transmission of droplet-borne and vector-borne, but not in waterborne diseases [112,113]. As almost 80% of all illnesses and deaths in low and middle-income countries are linked to poor water and sanitation conditions, further evidence-based and scientifically-rigorous studies should be conducted to better inform public health interventions in these contexts where financial and material resources are lacking [117]. The scientific merits of such alternatives should, therefore, be further evaluated and used to build effective strategies in regions that experience physical and economic water scarcity [6].

Fourth, there is inconsistency in the recommendations by research institutions for certain preventive interventions between research institutions. For handwashing interventions, the time required for washing to ensure proper hand hygiene was not specified in most studies [19,20,35,38,42,45,46,47,60,61,68,72,73,78,79,80,104,105]. On the other hand, while the WHO defines improved sanitation as better access to sanitation facilities [114], many of the reviewed studies did not specify what measures can be put in place in a household to achieve improved sanitation. There is also lacking evidence in the ways to maintain appropriate use and cleanliness of household and community facilities. This creates challenges in assessing the competitiveness of results.

Fifth, there is little evidence on the efficacy of chemoprophylaxis against WBD. Only one study included prophylactic supplements in their intervention, where a diarrhea pack with water purification sachet was distributed within the community [56]. Comparative evaluation for variation of preventive interventions, such as different types of prophylactic supplements and types of water storage containers are useful in the planning of cost-effective interventions and should be implemented in future studies. The use of the more economical regular soap is now favored in most handwashing campaigns as similar reduction in diarrheal incidence has been observed with the use of regular soap and anti-bacterial soap [68,72]. Due to the search strategy and key words used, vaccination was not identified as an intervention. However, it must be acknowledged immunization has been regarded as one of the most effective primary prevention methods against viral illnesses with observed effects in food-borne and vector-borne diseases [111,112]. Vaccines against typhoid, hepatitis A and cholera are recommended by the WHO to travelers visiting areas of increased WBD risks [118]. Cholera vaccination is also included in routine childhood vaccination programs in many countries worldwide where risk is high, although the high costs of procurement, delivery, and program implementation, coupled with gaps in community education and awareness, are barriers to vaccine delivery in low-income countries where WBD is most prevalent [115].

Sixth, there was limited evidence in comparative evaluation for variations of primary preventive interventions, such as efficacy of the different water storage containers, or different materials to maintain household cleanliness. Strengthening the available evidence in the above-mentioned areas will allow development of strategies for protecting against WBDs in low-resource settings.

This study summarized the most common eight primary prevention interventions identified in WASH-related literature and the strengths and limitations of their implementation to improve Health-EDRM outcomes in low-resource communities. There is value in subsequent studies assessing the risks of WASH at multiple levels as pertaining to these interventions through a number of alternative frameworks, including the WASH cluster strategic operations framework and other ecological models.

### 4.4. Study Strengths and Limitations

There are some limitations to this review. The review excluded non-English-based literature, non-electronically accessible civilian-published literature, grey literature or any publications before 2000. The review also excluded annual reports from specialized organizations, United Nations reports, or reports by national governments. The eight preventative interventions identified in this review do not constitute all of the non-pharmaceutical preventative behavior that is available in the mitigation of WBD. Moreover, this review has not disaggregated findings by pathogen, for example difference in efficacy of interventions between viral, bacterial, and parasitic diseases. This area warrants further research, in order to review predictive success of interventions across different areas with particular disease patterns.

Despite the limitations, this review was able to identify valuable behavioral interventions for the planning and implementation of health policies that prevent water-borne biological hazards. Preparedness in communities facing specific vulnerabilities could be reinforced through multi-faceted and multi-sectoral collaboration, with an emphasis on four key areas (risk understanding, governance, preparedness and resilience) as suggested by the primary prevention model for disaster risk reduction in the Sendai Framework for Disaster Risk Reduction [12].

## 5. Conclusions

WBD-associated health risks will remain an ongoing biological hazard to the rapidly globalized world, which highlights the importance of sustainable strategies. In order to meet the SDGs by 2030 [16], multi-sectoral, multi-level capacity building will be needed for sustainable health-EDRM practices, with research for the effectiveness of alternative methods to WBD prevention in low resource settings. The implementation of policies such as early warning systems to inform the associated health risks of seasonal outbreaks and community education that focuses on early symptom identification with subsequent health-seeking behaviors could allow for better prevention and control of unexpected outbreaks. Such policies would also be beneficial in the case of the recent COVID-19 pandemic as low-resource communities are more likely to be affected by the pandemic. Evidence-based research must be translated into feasible and effective actions for disaster risk mitigation and risk reduction.

## Figures and Tables

**Table 1:** The Oxford Centre for Evidence-Based Medicine (OCEBM) 2009 Levels of Evidence [17].

Level	Therapy/Prevention, Etiology/Harm
1A	Systematic Review (SR) (with homogeneity of randomized controlled trials (RCTs)
1B	Individual RCT (with narrow confidence interval)
1C	All or None
2A	SR (with homogeneity) of cohort studies
2B	Individual cohort study (including low quality RCT; e.g., <80% follow-up)
2C	“Outcomes” research; ecological studies
3A	SR (with homogeneity) of case control studies
3B	Individual case control study
4	Case series (and poor-quality cohort and case control studies)
5	Expert opinion without explicit critical appraisal, or based on physiology, bench research or “first principles”

**Table 2:** Overview of Health-EDRM Primary Prevention Approaches against Waterborne Diseases in the reviewed articles, categorized by the Levels of Evidence based upon Oxford Centre for Evidence-Based Medicine (OCEBM) criteria [17]. (Please see Table S1 for details).

Category	Primary Preventive Interventions	Number of Referenced Articles Per OCEBM Categorization Level										
		1a	1b	1c	2a	2b	2c	3a	3b	4	5	Total
Personal Interventions	Handwashing	4	4	1	1	3	1	0	1	4	1	20
	Prophylactic Supplements	0	1	0	0	0	0	0	0	0	0	1
Household Interventions	Water treatment	5	34	0	4	8	4	0	0	8	2	65
	Household safe water storage	1	8	0	2	3	1	0	0	3	2	20
	Household Cleanliness	4	0	0	1	1	1	0	1	3	0	11
	Household Waste Disposal	4	0	0	0	2	0	0	1	3	0	10
Community Interventions	Community Infrastructure	1	3	0	0	3	1	0	1	3	1	13
	Community Education	2	7	0	1	5	2	0	1	4	0	22
Total		21	57	1	9	25	10	0	5	28	6	162 ^1^
Key: Number of referenced articles reviewed per category, per intervention. 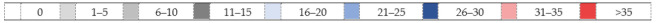												

^1^ Of the 88 publications reviewed, some included findings on more than one prevention measure, and are counted more than once.

**Table 3:** Personal protection practices as primary preventive interventions against WBDs: regular handwashing and intake of prophylactic supplements.

Parameters	Regular Handwashing	Prophylactic Supplements
Risk	Waterborne pathogens such as bacteria, viruses and parasites can be transmitted as one touches the eyes, nose or mouth after contacting contaminated water sources without adequate handwashing [106] Children are at risk of parasitic infections transmitted from the household environment if their caregivers do not practice adequate handwashing [47]. Approximately three billion people worldwide do not practice regular handwashing due to lack of access of soap and water, with higher incidence of diarrheal diseases in such population [107]	Dehydration is the most severe threat posed by diarrheal diseases, as water and electrolytes are lost through liquid stools, vomit and sweat. This could be life-threatening in severe cases where losses of electrolytes are not replaced [2] Zinc supplementation along with oral rehydration solution (ORS) has emerged as a potent approach in WBD management: zinc strengthens gut lining and reduces severity, whereas ORS replenishes electrolytes and rehydrates dehydrated individual [56]
Behavioral Change	Handwashing, with or without soap, in clean and running water at regular intervals to reduce the risk of contracting of WBD [19,20,35,38,42,45,46,47,60,61,68,72,73,78,79,80,104,105] Handwashing at vital times such as prior to food preparation and after toilet use to prevent transmission of waterborne pathogens via fecal–oral route [21,46]	Oral intake of zinc and oral rehydration salt to prevent and manage diarrheal illness by averting dehydration [56]
Co-benefits	Effective in reducing number of days with diarrhea in severely malnourished children [71] Reduces occurrence of other diseases such as respiratory infection [68,78], skin infections [68], and nutritional deficiency [47] Effective at preventing contraction of other diseases in HIV-infected children, regardless of anti-viral regimen [45] Visually cleaner hands [47]	Reduces antibiotics use in management of WBD [56] Reduces WBD associated hospitalization [56]
Enabling Factors	Access to clean water [19,49,60,61,80,105] Access to soap [104] with no difference in incidence of diarrhea between households with plain soap compared to antibacterial soap [20,69,72] Education: increase awareness of the needs and benefits of handwashing can further promote behavioral change [38,47,73,78,80] Financial support: sufficient funding to roll out hand hygiene interventions in schools with distribution of resources [78]	Education: understanding the benefits of supplements with appropriate consumption and dosage [56] Baseline water quality: purification sachets so prophylactic supplements can be taken with clean water to maximize effectiveness [56]
Limiting Factors	Distance of facilities: decrease in hand washing behavior when sanitation facilities are placed at a further distance [35] Ways of transmission: multiple pathways for ingestion of faecal pathogens and no significant difference has been found in the amount of ingested pathogens by children despite water, sanitation and hygiene interventions (WASH), as *E. coli* was still found on food [108] Socioeconomic status: poorer households are less able to adapt hand washing behavior rapidly [61] Unsustainable behavior: lack of health impact outside intervention period due to unsustained adaptation of behavioral change [76]	Access to prophylactic supplements [56]
Alternatives for resource-poor settings	Use of alcohol sanitizers Handwashing with ash, mud, soil with or without water which could inactivate and rub away pathogens [20]	Consumption of water-rich fruits and vegetables to prevent dehydration [109]
Strength of evidence	Beneficial effect of handwashing with soap (dependent on access) is consistent across various study designs, however, only few randomized control trials (RCT) compared to other interventions so strength of evidence is relatively weak [68,72,73,79,104] No additional reduction in diarrhea incidence when combining handwashing with water treatment intervention [60]	Only one study was identified that reported the association between increase in uptake of ORS and zinc supplements and lower prevalence of diarrhea [56]

**Table 4:** Household practices as primary preventive interventions against WBDs: household water treatment and household water storage.

Parameters	Household Water Treatment	Household Water Storage
Risk	Water contains many impurities and can be easily contaminated by harmful chemicals and waterborne pathogens (viruses, bacteria and parasites), which can lead to water-related diseases and other serious health issues if left untreated [7] Diarrhea incidence is positively associated with consumption of untreated and unsafe water [26,29,83,85,90] Boiling water is insufficient in killing all waterborne microbes and other new-age contaminants, and thus higher risks of diarrhea compared to other water treatment [29] Risk of recontamination during the process from water collection to consumption, point-of use treatment is therefore important to maintain health benefits from improved supply [100]	Water is subject to frequent and extensive microbial contamination during collection, transport and storage, as waterborne pathogens can still enter and propagate after the point of collection [31,43] Risk of regrowth of waterborne pathogens during unsafe storage of water contributes to challenges in maintaining clean water quality at point of consumption [37,41,55] Improving household drinking water quality through safe storage is protective against diarrheal disease [31,42,57,62,64,87]
Behavioral Change	Water handling: solar water disinfection (SODIS) [22,25,75,81,82,92,96,97,98], boiling [29,75] Use of chemical treatments: disinfection products [21,25,45,51,56,57,58,60,61,62,64,65,69,70,83,87,89,91,93,95,99,102,104,105], chlorination [29,30,35,42,44,59,63,66,71,75,84,100,102] Use of filtration system: LifeStraw filters [25,26,31,37,41,47,74,76], Biosand [25,31,40,46,52,53,86], ceramic filter [25,31,85,88,90,94,101], PointONE filter [48,50], UV disinfection system [55]	Use of household cisterns to collect rainwater from rooftops could provide solution to water quality and scarcity issues, and households with cisterns had significantly lower 30-day period prevalence of diarrhea than those without [36] Use of water storage containers: clay pots [30], jerry cans [41] Use of water storage vessels [45,62] Covering of water storage containers with lid [30,41,43,62,69]
Co-benefits	Beneficial effects in child development: prevention of malnutrition and increase in median height for age after SODIS (key health outcomes for children under 12) [92] Increased savings: not having to buy other resources to clean water and medical expenses [26,48,59] Protective against diarrhea in HIV-positive population [37,41,63,65,69,103] Improved drinking water quality [52,53,54,71,75,83,85,86,90,94] Reduce incidence of childhood acute respiratory infection with use of higher efficiency biomass cookstoves compared to use of open fire in boiling water [74]	Protective against diarrhea in HIV-positive population [37,41,62,65,69] Protective against vector-borne diseases, as insects are unable to access and breed in water stored in closed container Improved water quality [64]
Enabling Factors	Availability and access to water treatment products [25,26,41,94,97] Compliance to water treatment regime [26,29,41,70,74,81,82] Water storage system: minimize risk of recontamination at point-of-consumption [37,41] Education: skills to repair of malfunctioning devices [26,77] Availability of heat source and kerosene for boiling [29] Availability of bright sunlight for SODIS [22,25,75,81,82,92,96,97,98]	Availability and access to water storage containers and facilities Compliance to water storage regime: social marketing campaigns and support from management committees to ensure participation and adherence from households [30] Water treatment combination: water storage system improvements that resulted in positive health benefits were often combined with use of water filter [31,41,42,45,51,55,58,59,61,62,63,64,65,69,87,89,102]; no positive health benefit in clay pots were observed without water treatment [30]
Limiting Factors	Age of children: young children are more exposed to pathogens as they play in a contaminated environment, and intake of supplementary fluids prepared with untreated water outside of weaning period [22,84] Socioeconomic status: wealthy and more educated households are able to afford water treatment products and adapt to water treatment behavior more quickly [61,102] Exposure to untreated water sources outside of household [22,55,66,81] Seasonal variability: differences in precipitation and temperature could influence concentration of microorganisms present in water [52] Poor product acceptability: unpleasant taste associated with chlorination treatment [29] Marital status: adoption of SODIS linked to status [81] Cultural beliefs: some communities believe that boiling water is sufficient in preventing WBD as it has been heavily promoted for decades, and are therefore reluctant to adapt other treatments [29]	Socioeconomic status: wealthy households are able to adapt water treatment behavior more quickly [61,102] Suitable and appropriate design of storage containers: less compliance with unpopular designs, but increase in the use of storage containers with a more practical design despite lower effectiveness compared to other storage methods [43]
Alternatives for resource-poor settings	Point of use filtration in areas where water infrastructure facilities are not improved [25] SODIS is adopted in low-income households as they cannot afford filters, reduction in diarrhea incidence is still observed although less compared to the use of filter [25] Use of bleach in low-income households as they cannot afford flocculant disinfectant, reduction in diarrhea incidence is still apparent although less compared to use of disinfectant [51,60] Bottled drinking water: similar reduction in diarrhea incidence when compared to water treatment [95]	Use bottled drinking water where possible
Strength of evidence	Interventions with aims to improve microbial quality of water are significantly associated with effective prevention of diarrheal diseases, as seen in many RCTs [22,25,41,44,48,50,51,52,53,55,56,58,59,60,64,69,70,71,74,82,83,84,85,86,87,88,89,90,92,93,94,96,97,98,99,101,102,104] Effectiveness of water treatment is not enhanced when combined with other interventions such as improved sanitation and basic hygiene practice [25,50,60]	Community-based interventions combining treatment and storage are effective in the reduction of diarrhea incidence; however there are few RCT-based systematic reviews [58,87] Only 2 out of 20 studies investigated the beneficial effect of water storage alone [36,43] Studies into water storage combined with other intervention have shown that safe storage is most effective when coupled with water treatment or filtration [31,41,42,45,51,55,58,59,61,62,63,64,65,69,87,89,102]

**Table 5:** Household practices as primary preventive interventions against WBDs (continued): household cleanliness and household waste management.

Parameters	Household Cleanliness	Household Waste Management
Risk	Waterborne pathogens can persist on surfaces for a few days. Hand-contact surfaces, food-contact surfaces and household linens can be responsible for WBD transmission through viruses, bacteria and parasites. Improvements in sanitation achieved by increased cleanliness is associated with decreased risks of diarrhea [47,58,77,104,105] High concentration of pathogens can be found in certain mud floors in rural areas, as they are painted with animal dung, which accounts for the high prevalence of diarrhea observed in those living in households with mud [105]	Ingestion and exposure to human waste is associated with diarrhea and other WBD; interventions aimed at improving excreta disposal have found to be protective [24] Shared sanitation facilities tend to be dirtier than private facilities, can be easily contaminated with waterborne pathogens, and are therefore associated with higher risks of moderate-to-severe diarrhea [20,47] 17% of rural population remain without access to a toilet or latrine, which leads to practice of open defecation and unsafe faecal disposal, contributing to sustained increase of diarrhea incidence [34] Children in households with simple pit latrine have 7 times higher odds of intestinal parasitic infection than those with water-sealed latrines [47]
Behavioral Change	Maintain cleanliness of household sanitation facilities [19,58,77] Lay concrete floor in household [105]	Improve excreta disposal by constructing facilities to encourage closed defecation: latrines, borehole latrines, household flush toilets, piped water system, private water sealed toilets [24,34,58] Drain contaminated, stagnant water [19]
Co-benefits	Reduces WBD associated hospitalization [19,62] Improves overall hygiene and standard of living	Reduce WBD-associated hospitalization [20] Reduce risk of fever with drainage of stagnant water [19] Reduce incidence of vector-borne diseases by draining stagnant water, where vectors breed [19]
Enabling Factors	Availability and access to cleaning products Sustainable behavior: small scale monitoring required at household levels for long term behavioral change [28] Education: appropriate sanitation practice [77]	Access to household building materials for construction [20,34,77] Availability of spaces in households to build private sanitation infrastructures to improve waste management [20] Education to maximize facility usage and knowledge on how to build sanitation infrastructure [20,34,77]
Limiting Factors	Cultural practice: painting of mud floors with animal dung remains widespread in rural community [47] Good hygiene practice: sanitation coverage alone is not adequate to improve hygiene outcomes so therefore should be combined with other interventions [28,34,87] Affordability to lay concrete floor	Neighbors: household members with improved sanitation may still be exposed to waterborne pathogens if their neighbors have no improved sanitation due to close proximity [20,34,77] Affordability for construction: household sewer connection was associated with greater reduction in diarrhea compared to other household sanitation facilities [58]
Alternatives for resource-poor settings	Use water to clean instead of cleaning products Lay low-cost earthen adobe floor to replace dirt floor [110]	Minimize the number of households that share the facilities [20,47]
Strength of evidence	No studies mentioned ways of implementation to maintain cleanliness (e.g., use and effectiveness of cleaning products) Strong evidence for association between improvements in sanitation and decreased risks of diarrhea derived from systematic review of RCTs, however only 2 systematic reviews were identified [28,39,58] Only one study identified showing the association between clean floor and WBD [47]	Intervention studies aimed at improving disposal excreta have found to be protective against diarrhea; however only a few studies in multiple settings were identified and many of them combined other sanitation interventions [19,20,30,34,39,58,104]

**Table 6:** Community practices as primary preventive interventions against WBDs: community infrastructure and community education.

Parameters	Community Infrastructure	Community Education
Risk	Poorly managed or designed infrastructure increases the risk of contamination of water by chemicals and pathogens (viruses, bacteria, parasites); improvements reduce symptoms and incidence of WBD [38,67,87] Lack of water infrastructure in the community does not allow regular water supply and thus water scarcity, which could contribute to WBD burden [23,28,77]	Educational interventions have important and sustainable health benefits in reducing rate of diarrheal illnesses caused by variety of agents, bacteria, viruses or parasites [33,49,62,69,79,80] Increase in risk factors for the contraction of infectious diseases without appropriate knowledge on proper hygiene [39]
Behavioral Change	Drilling or rehabilitating boreholes [23,27,87] Sinking of wells [27] Building communal water stations [35,38,87] Building piped water supply in communities [47,54,77,95] Developing a functional and closed sewer system [67]	Community participation in WASH interventions, meetings and/or education campaigns [18,21,27,28,32,33,77,79,80,102,103,105]
Co-benefits	Promote behavioral change: increased number of households with hygiene enabling-facilities (rubbish pits, pot racks) [27] along with increased handwashing and soap use [38] Economic benefits: increased number of customers in business with installation of tippy-taps [38]	Prevention of reinfection by intestinal parasites [32] Following education, communities were less likely to report unpleasant odor from treated water [18] Education allows communities to manage own water quality [33,49] Teacher-training shown to lead to pupil’s improvements [35,80] Decreased in medical costs and inability to work [62] Effective in preventing diarrhea in HIV-positive population [64,65,69,103]
Enabling Factors	Use of community infrastructures [54] Appropriate hygiene behavior: availability of water alone without other interventions may not influence incidence of WBD [23,34,35,39,87] Availability of resources and space for construction and maintenance of community infrastructure [35,38,67] Education: understand the importance of improved water supply and the purpose of facilities to maximize usage [77]	Access to resources for full adaptation of suggested behavioral change (e.g., soap, filters, sanitation) [34,39] Motivation for villagers to attend educational interventions [102] Properly-designed intervention: trained personnel to deliver health messages, dissemination of information correctly and effectively [42] Appropriate communication: intervention delivered in a culturally-sensitive manner [56,102] Financial support: sufficient funding to roll out educational campaigns [78]
Limiting Factors	Inadequate funding from NGOs and government for WASH interventions as costs are higher compared to health and hygiene interventions [27,67] Distance to water source: increase risk of contamination during transportation from water source to point-of-consumption, and reduce quantity of water from loss during transportation [38,99] Interruption to use of facilities: households with interruption to water supply had 2.87 higher odds of diarrhea [47]	Underlying scepticism about waterborne disease transmission: villagers believed that WBD outbreak started because of ancestral curse or witchcraft [21] and the lack of health risks in pathogens [32] Economic hardship: communities had good knowledge but unable to adapt behavioral change due to unaffordability [27]
Alternatives for resource-poor settings	Using bottled water when possible [95] Harvest rainwater and stormwater, or reuse water, to be treated and used along with other WBD interventions [30]	Emphasize the importance of handwashing in educational campaigns as it is less costly compared to other interventions (e.g., filter use) [27,79] Higher reduction of diarrhea incidence is seen in children receiving intervention with education and handwashing compared to those with education and other interventions [39,50,79,80,99,105]
Strength of evidence	Low strength of evidence due to low intervention uptake which confers difficulty in evaluating the impacts of intervention [54]	Significant association between education intervention and reduction in diarrheal incidence as seen in RCTs [39,56,79,102]

## Data Availability

Not available.

## Supplementary Materials

The following are available online at https://www.mdpi.com/article/10.3390/ijerph182312268/s1, Table S1: Relevant interventions, study design, relevant key findings, and conclusion of each utilized reference, Table S2: Coding for each type of intervention in Table S1.

## Appendix A

Pathogens transmitted through drinking water are diverse in causative agent, characteristics, and health significance. Table A1 shows pathogens that are globally significant for water safety and supply management [119].

**Table A1:** Pathogens associated with water-borne diseases, by global significance of incidence and disease severity.

Incidence and Severity	Pathogen	Organism	Associated Diseases
High	*Burkholderia*	Bacteria	Melioidosis
	*Campylobacter*	Bacteria	Campylobacteriosis
	*Escherichia coli*	Bacteria	E. Coli
	*Francisella*	Bacteria	Tularemia
	*Legionella*	Bacteria	Legionnaires’ disease
	*Salmonella*	Bacteria	Salmonella
	*Shigella*	Bacteria	Shigella
	*Vibrio*	Bacteria	Cholera
	Caliciviridae	Virus	Calciviral infection
	Hepeviridae	Virus	Hepatitis
	Picornaviridae	Virus	Poliovirus
	Reoviridae	Virus	Rotavirus
	*Acanthamoeba*	Protozoa	Acanthamoeba keratitis
	*Cryptosporidium*	Protozoa	Cryptosporidiosis
	*Cyclospora*	Protozoa	Cyclospora infection
	*Entamoeba*	Protozoa	Amebiasis
	*Giardia*	Protozoa	Giardiasis
	*Naegleria*	Protozoa	Naegleria infection
	*Dracunculus*	Helminth	Guinea-worm disease
Moderate	Adenoviridae	Virus	Adenovirus infection
	Astroviridae	Virus	Astrovirus infection
Low	Mycobacteria	Bacteria	Mycobacteria infection

## References

[1] World Health Organization (2015). Infectious Diseases. Health in 2015: From MDGs to SDGs. https://cdn.who.int/media/docs/default-source/gho-documents/health-in-2015-mdgs-to-sdgs/health-in-2015-from-mdgs-to-sdgs.pdf?sfvrsn=8ba61059_2.

[2] World Health Organization Diarrhoeal Disease, 2 May 2017. https://www.who.int/news-room/fact-sheets/detail/diarrhoeal-disease.

[3] Patwari A.K. (1999). Diarrhoea and Malnutrition Interaction. Indian J. Pediatr..

[4] Sun R., An D., Lu W., Shi Y., Wang L., Zhang C., Zhang P., Qi H., Wang Q. (2016). Impacts of a Flash Flood on Drinking Water Quality: Case Study of Areas Most Affected by the 2012 Beijing Flood. Heliyon.

[5] Li S., Elliott S.J. (2016). Facilitators and Barriers to Effective Water and Sanitation Interventions for Characterizing Shigellosis Incidence in Jiangsu, China. Procedia Environ. Sci..

[6] Giordano M., Barron J., Ünver O., Campanhola C., Pandey S. (2019). Chapter 5-Water Scarcity and Challenges for Smallholder Agriculture. Sustainable Food and Agriculture.

[7] Macy J.T., Quick R.E. (2010). Transmission and Prevention of Water-Related Diseases. Water Health.

[8] Walker D.B., Baumgartner D.J., Gerba C.P., Fitzsimmons K., Brusseau M.L., Pepper I.L., Gerba C.P. (2019). Chapter 16-Surface Water Pollution. Environmental and Pollution Science.

[9] United Nations Clean Water and Sanitation: Why It Matters. https://www.un.org/sustainabledevelopment/wp-content/uploads/2018/09/Goal-6.pdf.

[10] Ziv T., Heymann A.D., Azuri J., Leshno M., Cohen D. (2011). Assessment of the Underestimation of Childhood Diarrhoeal Disease Burden in Israel. Epidemiol. Infect..

[11] World Health Organization (2019). Health Emergency and Disaster Risk Management Framework. https://www.who.int/hac/techguidance/preparedness/health-emergency-and-disaster-risk-management-framework-eng.pdf.

[12] World Health Organization (2015). Sendai Framework for Disaster RIsk Reduction 2015–2030. https://www.preventionweb.net/files/43291_sendaiframeworkfordrren.pdf.

[13] Chan E.Y.Y., Shaw R. (2020). Public Health and Disasters: Health Emergency and Disaster Risk Management in Asia.

[14] World Health Organization (2010). Improving Healthcare: Individual Interventions. Global Status Report on Noncommunicable Diseases 2010. https://www.who.int/nmh/publications/ncd_report_full_en.pdf.

[15] Piper J.D., Chandna J., Allen E., Linkman K., Cumming O., Prendergast A.J., Gladstone M.J. (2017). Water, Sanitation and Hygiene (WASH) Interventions: Effects on Child Development in Low- and Middle-Income Countries. Cochrane Database Syst. Rev..

[16] United Nations (2020). The Sustainable Development Goals Report. https://unstats.un.org/sdgs/report/2020/The-Sustainable-Development-Goals-Report-2020.pdf.

[17] OCEBM Levels of Evidence Working Group. “The Oxford Levels of Evidence 2”. Oxford Centre for Evidence-Based Medicine. https://www.cebm.ox.ac.uk/resources/levels-of-evidence/ocebm-levels-of-evidence.

[18] Ananga E.O., Njoh A.J., Pappas C., Ananga G.O. (2017). Examining the Relationship Between Community Participation and Water Handling Hygiene Practices in the Informal Neighborhoods of Kisumu, Kenya. Habitat. Int..

[19] Anthonj C., Githinji S., Kistemann T. (2018). The Impact of Water on Health and Ill-Health in a Sub-Saharan African Wetland: Exploring Both Sides of the Coin. Sci. Total Environ..

[20] Baker K.K., O’Reilly C.E., Levine M.M., Kotloff K.L., Nataro J.P., Ayers T.L., Farag T.H., Nasrin D., Blackwelder W.C., Wu Y. (2016). Sanitation and Hygiene-Specific Risk Factors for Moderate-to-Severe Diarrhea in Young Children in the Global Enteric Multicenter Study, 2007–2011: Case-Control Study. PLoS Med..

[21] Bennett S.D., Lowther S.A., Chingoli F., Chilima B., Kabuluzi S., Ayers T.L., Warne T.A., Mintz E. (2018). Assessment of Water, Sanitation and Hygiene Interventions in Response to an Outbreak of Typhoid Fever in Neno District, Malawi. PLoS ONE.

[22] Bitew B.D., Gete Y.K., Biks G.A., Adafrie T.T. (2018). The Effect of SODIS Water Treatment Intervention at the Household Level in Reducing Diarrheal Incidence among Children under 5 Years of Age: A Cluster Randomized Controlled Trial in Dabat District, Northwest Ethiopia. Trials.

[23] Cha S., Kang D., Tuffuor B., Lee G., Cho J., Chung J., Kim M., Lee H., Lee J., Oh C. (2015). The Effect of Improved Water Supply on Diarrhea Prevalence of Children under Five in the Volta Region of Ghana: A Cluster-Randomized Controlled Trial. Int. J. Environ. Res. Public Health.

[24] Clasen T.F., Bostoen K., Schmidt W.P., Boisson S., Fung I.C., Jenkins M.W., Scott B., Sugden S., Cairncross S. (2010). Interventions to Improve Disposal of Human Excreta for Preventing Diarrhoea. Cochrane Database Syst. Rev..

[25] Clasen T.F., Alexander K.T., Sinclair D., Boisson S., Peletz R., Chang H.H., Majorin F., Cairncross S. (2015). Interventions to Improve Water Quality for Preventing Diarrhoea. Cochrane Database Syst. Rev..

[26] De Ver Dye T., Apondi R., Lugada E., Kahn J.G., Sandiford-Day M.A., Dasbanerjee T. (2011). A Qualitative Assessment of Beliefs, Attitudes, and Behaviors Related to Diarrhea and Water Filtration in Rural Kenya. Am. J. Public Health.

[27] Demberere T., Muyambo M., Mutengu S., Ncozana T., Manyeruke N. (2014). An Analysis of the Effectiveness of WASH Interventions in Relation to Diarrhoeal Diseases in Chipinge District, Zimbabwe. Phys. Chem. Earth.

[28] Dey N.C., Parvez M., Islam M.R., Mistry S.K., Levine D.I. (2019). Effectiveness of a Community-Based Water, Sanitation, and Hygiene (WASH) Intervention in Reduction of Diarrhoea among Under-Five Children: Evidence from a Repeated Cross-Sectional Study (2007–2015) in Rural Bangladesh. Int. J. Hyg. Environ. Health.

[29] Fagerli K., Trivedi K.K., Sodha S.V., Blanton E., Ati A., Nguyen T., Delea K.C., Ainslie R., Figueroa M.E., Kim S. (2017). Comparison of Boiling and Chlorination on the Quality of Stored Drinking Water and Childhood Diarrhoea in Indonesian Households. Epidemiol. Infect..

[30] Garrett V., Ogutu P., Mabonga P., Ombeki S., Mwaki A., Aluoch G., Phelan M., Quick R.E. (2008). Diarrhoea Prevention in a High-Risk Rural Kenyan Population through Point-of-use Chlorination, Safe Water Storage, Sanitation, and Rainwater Harvesting. Epidemiol. Infect..

[31] Clasen T.F., Selendy J.M.H. (2019). Household Water Treatment and Safe Storage in Low-Income Countries. Water and Sanitation-Related Diseases and the Changing Environment: Challenges, Interventions, and Preventive Measures.

[32] Gungoren B., Latipov R., Regallet G., Musabaev E. (2007). Effect of Hygiene Promotion on the Risk of Reinfection Rate of Intestinal Parasites in Children in Rural Uzbekistan. Trans. R. Soc. Trop. Med. Hyg..

[33] Hunter P.R., Ramírez Toro G.I., Minnigh H.A. (2010). Impact on Diarrhoeal Illness of a Community Educational Intervention to Improve Drinking Water Quality in Rural Communities in Puerto Rico. BMC Public Health.

[34] Kamara J.K., Galukande M., Maeda F., Luboga S., Renzaho A.M.N. (2017). Understanding the Challenges of Improving Sanitation and Hygiene Outcomes in a Community Based Intervention: A Cross-Sectional Study in Rural Tanzania. Int. J. Environ. Res. Public Health.

[35] La Con G., Schilling K., Harris J., Person B., Owuor M., Ogange L., Faith S., Quick R. (2017). Evaluation of Student Handwashing Practices During a School-Based Hygiene Program in Rural Western Kenya, 2007. Int. Q. Community Health Educ..

[36] Marcynuk P.B., Flint J.A., Sargeant J.M., Jones-Bitton A., Brito A.M., Luna C.F., Szilassy E., Thomas M.K., Lapa T.M., Perez E. (2013). Comparison of the Burden of Diarrhoeal Illness among Individuals with and without Household Cisterns in Northeast Brazil. BMC Infect. Dis..

[37] Mbakaya B.C., Kalembo F.W., Zgambo M. (2019). Community-Based Interventions for Preventing Diarrhoea in People Living with HIV in Sub-Sahara Africa: A Systematic Review. Malawi Med. J..

[38] Mbakaya B.C., Kalembo F.W., Zgambo M. (2020). Use, Adoption, and Effectiveness of Tippy-Tap Handwashing Station in Promoting Hand Hygiene Practices in Resource-Limited Settings: A Systematic Review. BMC Public Health.

[39] McDonald E., Bailie R., Brewster D., Morris P. (2008). Are Hygiene and Public Health Interventions Likely to Improve Outcomes for Australian Aboriginal Children Living in Remote Communities? A Systematic Review of the Literature. BMC Public Health.

[40] Moropeng R.C., Budeli P., Mpenyana-Monyatsi L., Momba M.N.B. (2018). Dramatic Reduction in Diarrhoeal Diseases through Implementation of Cost-Effective Household Drinking Water Treatment Systems in Makwane Village, Limpopo Province, South Africa. Int. J. Environ. Res. Public Health.

[41] Peletz R., Simunyama M., Sarenje K., Baisley K., Filteau S., Kelly P., Clasen T. (2012). Assessing Water Filtration and Safe Storage in Households with Young Children of HIV-Positive Mothers: A Randomized, Controlled Trial in Zambia. PLoS ONE.

[42] Ramesh A., Blanchet K., Ensink J.H., Roberts B. (2015). Evidence on the Effectiveness of Water, Sanitation, and Hygiene (WASH) Interventions on Health Outcomes in Humanitarian Crises: A Systematic Review. PLoS ONE.

[43] Roberts L., Chartier Y., Chartier O., Malenga G., Toole M., Rodka H. (2001). Keeping Clean Water Clean in a Malawi Refugee Camp: A Randomized Intervention Trial. Bull. World Health Organ..

[44] Solomon E.T., Robele S., Kloos H., Mengistie B. (2020). Effect of Household Water Treatment with Chlorine on Diarrhea among Children under the Age of Five Years in Rural Areas of Dire Dawa, Eastern Ethiopia: A Cluster Randomized Controlled Trial. Infect. Dis. Poverty.

[45] Sugar N.R., Schilling K.A., Kim S., Ahmed A., Ngui Muyanga D., Sivapalasingam S., Quick R. (2017). Integrating Household Water Treatment, Hand Washing, and Insecticide-Treated Bed Nets Into Pediatric HIV Care in Mombasa, Kenya: Impact on Diarrhea and Malaria Risk. J. Acquir. Immune Defic. Syndr..

[46] Uwimpuhwe M., Reddy P., Barratt G., Bux F. (2014). The Impact of Hygiene and Localised Treatment on the Quality of Drinking Water in Masaka, Rwanda. J. Environ. Sci. Health A Tox. Hazard. Subst. Environ. Eng..

[47] Shrestha A., Six J., Dahal D., Marks S., Meierhofer R. (2020). Association of Nutrition, Water, Sanitation and Hygiene Practices with Children’s Nutritional Status, Intestinal Parasitic Infections and Diarrhoea in Rural Nepal: A Cross-Sectional Study. BMC Public Health.

[48] Tintle N., Van De Griend K., Ulrich R., Wade R.D., Baar T.M., Boven E., Cooper C.E.A., Couch O., Eekhoff L., Fry B. (2021). Diarrhea Prevalence in a Randomized, Controlled Prospective Trial of Point-Of-Use Water Filters in Homes and Schools in the Dominican Republic. Trop. Med. Health.

[49] Pawestri A.R., Thima K., Leetachewa S., Maneekan P., Deesitthivech O., Pinna C., Yingtaweesak T., Moonsom S. (2021). Seasonal Prevalence, Risk Factors, and One Health Intervention for Prevention of Intestinal Parasitic Infection in Underprivileged Communities on the Thai-Myanmar Border. Int. J. Infect. Dis..

[50] Lindquist E.D., George C.M., Perin J., Neiswender de Calani K.J., Norman W.R., Davis T.P., Perry H. (2014). A Cluster Randomized Controlled Trial to Reduce Childhood Diarrhea Using Hollow Fiber Water Filter and/or Hygiene-Sanitation Educational Interventions. Am. J. Trop. Med. Hyg..

[51] Reller M.E., Mendoza C.E., Lopez M.B., Alvarez M., Hoekstra R.M., Olson C.A., Baier K.G., Keswick B.H., Luby S.P. (2003). A Randomized Controlled Trial of Household-Based Flocculant-Disinfectant Drinking Water Treatment for Diarrhea Prevention in Rural Guatemala. Am. J. Trop. Med. Hyg..

[52] Stauber C.E., Ortiz G.M., Loomis D.P., Sobsey M.D. (2009). A Randomized Controlled Trial of the Concrete Biosand Filter and Its Impact on Diarrheal Disease in Bonao, Dominican Republic. Am. J. Trop. Med. Hyg..

[53] Fabiszewski de Aceituno A.M., Stauber C.E., Walters A.R., Meza Sanchez R.E., Sobsey M.D. (2012). A Randomized Controlled Trial of the Plastic-Housing Biosand Filter and Its Impact on Diarrheal Disease in Copan, Honduras. Am. J. Trop. Med. Hyg..

[54] McGuinness S.L., O’Toole J., Forbes A.B., Boving T.B., Patil K., D’Souza F., Gaonkar C.A., Giriyan A., Barker S.F., Cheng A.C. (2020). A Stepped Wedge Cluster-Randomized Trial Assessing the Impact of a Riverbank Filtration Intervention to Improve Access to Safe Water on Health in Rural India. Am. J. Trop. Med. Hyg..

[55] Gruber J.S., Reygadas F., Arnold B.F., Ray I., Nelson K., Colford J.M. (2013). A Stepped Wedge, Cluster-Randomized Trial of a Household UV-Disinfection and Safe Storage Drinking Water Intervention in Rural Baja California Sur, Mexico. Am. J. Trop. Med. Hyg..

[56] Habib M.A., Soofi S., Sadiq K., Samejo T., Hussain M., Mirani M., Rehmatullah A., Ahmed I., Bhutta Z.A. (2013). A Study to Evaluate the Acceptability, Feasibility and Impact of Packaged Interventions (“Diarrhea Pack”) for Prevention and Treatment of Childhood Diarrhea in Rural Pakistan. BMC Public Health.

[57] Gundry S., Wright J., Conroy R. (2004). A Systematic Review of the Health Outcomes Related to Household Water Quality in Developing Countries. J. Water Health.

[58] Wolf J., Prüss-Ustün A., Cumming O., Bartram J., Bonjour S., Cairncross S., Clasen T., Colford J.M., Curtis V., De France J. (2014). Assessing the Impact of Drinking Water and Sanitation on Diarrhoeal Disease in Low- and Middle-Income Settings: Systematic Review and Meta-Regression. Trop. Med. Int. Health.

[59] Sobsey M.D., Handzel T., Venczel L. (2003). Chlorination and Safe Storage of Household Drinking Water in Developing Countries to Reduce Waterborne Disease. Water Sci. Technol..

[60] Luby S.P., Agboatwalla M., Painter J., Altaf A., Billhimer W., Keswick B., Hoekstra R.M. (2006). Combining Drinking Water Treatment and Hand Washing for Diarrhoea Prevention, A Cluster Randomised Controlled Trial. Trop. Med. Int. Health.

[61] Luby S.P., Agboatwalla M., Hoekstra R.M., Rahbar M.H., Billhimer W., Keswick B.H. (2004). Delayed Effectiveness of Home-Based Interventions in Reducing Childhood Diarrhea, Karachi, Pakistan. Am. J. Trop. Med. Hyg..

[62] Migele J., Ombeki S., Ayalo M., Biggerstaff M., Quick R. (2007). Diarrhea Prevention in a Kenyan School through the Use of a Simple Safe Water and Hygiene Intervention. Am. J. Trop. Med. Hyg..

[63] Barzilay E.J., Aghoghovbia T.S., Blanton E.M., Akinpelumi A.A., Coldiron M.E., Akinfolayan O., Adeleye O.A., LaTrielle A., Hoekstra R.M., Gilpin U. (2011). Diarrhea Prevention in People Living with HIV: An Evaluation of a Point-Of-Use Water Quality Intervention in Lagos, Nigeria. AIDS Care.

[64] Quick R.E., Kimura A., Thevos A., Tembo M., Shamputa I., Hutwagner L., Mintz E. (2002). Diarrhea Prevention through Household-Level Water Disinfection and Safe Storage in Zambia. Am. J. Trop. Med. Hyg..

[65] Harris J.R., Greene S.K., Thomas T.K., Ndivo R., Okanda J., Masaba R., Nyangau I., Thigpen M.C., Hoekstra R.M., Quick R.E. (2009). Effect of a Point-Of-Use Water Treatment and Safe Water Storage Intervention on Diarrhea in Infants of HIV-Infected Mothers. J. Infect. Dis..

[66] Jensen P.K., Ensink J.H., Jayasinghe G., van der Hoek W., Cairncross S., Dalsgaard A. (2003). Effect of Chlorination of Drinking-Water on Water Quality and Childhood Diarrhoea in a Village in Pakistan. J. Health Popul. Nutr..

[67] Barreto M.L., Genser B., Strina A., Teixeira M.G., Assis A.M.O., Rego R.F., Teles C.A., Prado M.S., Matos S.M.A., Santos D.N. (2007). Effect of City-Wide Sanitation Programme on Reduction in Rate of Childhood Diarrhoea in Northeast Brazil: Assessment by Two Cohort Studies. Lancet.

[68] Luby S.P., Agboatwalla M., Feikin D.R., Painter J., Billhimer W., Altaf A., Hoekstra R.M. (2005). Effect of Handwashing on Child Health: A Randomised Controlled Trial. Lancet.

[69] Lule J.R., Mermin J., Ekwaru J.P., Malamba S., Downing R., Ransom R., Nakanjako D., Wafula W., Hughes P., Bunnell R. (2005). Effect of Home-Based Water Chlorination and Safe Storage on Diarrhea among Persons with Human Immunodeficiency Virus in Uganda. Am. J. Trop. Med. Hyg..

[70] Boisson S., Stevenson M., Shapiro L., Kumar V., Singh L.P., Ward D., Clasen T. (2013). Effect of Household-Based Drinking Water Chlorination on Diarrhoea among Children under Five in Orissa, India: A Double-Blind Randomised Placebo-Controlled Trial. PLoS Med..

[71] Pickering A.J., Crider Y., Sultana S., Swarthout J., Goddard F.G.B., Anjerul Islam S., Sen S., Ayyagari R., Luby S.P. (2019). Effect of In-Line Drinking Water Chlorination at the Point of Collection on Child Diarrhoea in Urban Bangladesh: A Double-Blind, Cluster-Randomised Controlled Trial. Lancet Glob. Health.

[72] Luby S.P., Agboatwalla M., Painter J., Altaf A., Billhimer W.L., Hoekstra R.M. (2004). Effect of Intensive Handwashing Promotion on Childhood Diarrhea in High-Risk Communities in Pakistan: A Randomized Controlled Trial. JAMA.

[73] Curtis V., Cairncross S. (2003). Effect of Washing Hands with Soap on Diarrhoea Risk in the Community: A Systematic Review. Lancet Infect. Dis..

[74] Kirby M.A., Nagel C.L., Rosa G., Zambrano L.D., Musafiri S., Ngirabega J.D., Thomas E.A., Clasen T. (2019). Effects of a Large-Scale Distribution of Water Filters and Natural Draft Rocket-Style Cookstoves on Diarrhea and Acute Respiratory Infection: A Cluster-Randomized Controlled Trial in Western Province, Rwanda. PLoS Med..

[75] Harshfield E., Lantagne D., Turbes A., Null C. (2012). Evaluating the Sustained Health Impact of Household Chlorination of Drinking Water in Rural Haiti. Am. J. Trop. Med. Hyg..

[76] Arnold B., Arana B., Mäusezahl D., Hubbard A., Colford J.M. (2009). Evaluation of a Pre-Existing, 3-Year Household Water Treatment and Handwashing Intervention in Rural Guatemala. Int. J. Epidemiol..

[77] Nanan D., White F., Azam I., Afsar H., Hozhabri S. (2003). Evaluation of a Water, Sanitation, and Hygiene Education Intervention on Diarrhoea in Northern Pakistan. Bull. World Health Organ..

[78] Mbakaya B.C., Lee P.H., Lee R.L. (2017). Hand Hygiene Intervention Strategies to Reduce Diarrhoea and Respiratory Infections among Schoolchildren in Developing Countries: A Systematic Review. Int. J. Environ Res Public Health.

[79] Ejemot R.I., Ehiri J.E., Meremikwu M.M., Critchley J.A. (2008). Hand Washing for Preventing Diarrhoea. Cochrane Database Syst. Rev..

[80] Hashi A., Kumie A., Gasana J. (2017). Hand Washing with Soap and WASH Educational Intervention Reduces Under-Five Childhood Diarrhoea Incidence in Jigjiga District, Eastern Ethiopia: A Community-Based Cluster Randomized Controlled Trial. Prev. Med. Rep..

[81] Graf J., Zebaze Togouet S., Kemka N., Niyitegeka D., Meierhofer R., Gangoue Pieboji J. (2010). Health Gains from Solar Water Disinfection (SODIS): Evaluation of a Water Quality Intervention in Yaoundé, Cameroon. J. Water Health.

[82] McGuigan K.G., Samaiyar P., du Preez M., Conroy R.M. (2011). High Compliance Randomized Controlled Field Trial of Solar Disinfection of Drinking Water and Its Impact on Childhood Diarrhea in Rural Cambodia. Environ. Sci. Technol..

[83] Crump J.A., Otieno P.O., Slutsker L., Keswick B.H., Rosen D.H., Hoekstra R.M., Vulule J.M., Luby S.P. (2005). Household Based Treatment of Drinking Water with Flocculant-Disinfectant for Preventing Diarrhoea in Areas with Turbid Source Water in Rural Western Kenya: Cluster Randomised Controlled Trial. BMJ.

[84] Mengistie B., Berhane Y., Worku A. (2013). Household Water Chlorination Reduces Incidence of Diarrhea among Under-Five Children in Rural Ethiopia: A Cluster Randomized Controlled Trial. PLoS ONE.

[85] Clasen T., Garcia Parra G., Boisson S., Collin S. (2005). Household-Based Ceramic Water Filters for the Prevention of Diarrhea: A Randomized, Controlled Trial of a Pilot Program in Colombia. Am. J. Trop. Med. Hyg..

[86] Tiwari S.S.K., Schmidt W.P., Darby J., Kariuki Z.G., Jenkins M.W. (2009). Intermittent Slow Sand Filtration for Preventing Diarrhoea among Children in Kenyan Households Using Unimproved Water Sources: Randomized Controlled Trial. Trop. Med. Int. Health.

[87] Clasen T., Schmidt W.P., Rabie T., Roberts I., Cairncross S. (2007). Interventions to Improve Water Quality for Preventing Diarrhoea: Systematic Review and Meta-Analysis. BMJ.

[88] Brown J., Sobsey M.D., Loomis D. (2008). Local Drinking Water Filters Reduce Diarrheal Disease in Cambodia: A Randomized, Controlled Trial of the Ceramic Water Purifier. Am. J. Trop. Med. Hyg..

[89] Doocy S., Burnham G. (2006). Point-Of-Use Water Treatment and Diarrhoea Reduction in the Emergency Context: An Effectiveness Trial in Liberia. Trop. Med. Int. Health.

[90] Clasen T.F., Brown J., Collin S.M. (2006). Preventing Diarrhoea with Household Ceramic Water Filters: Assessment of a Pilot Project in Bolivia. Int. J. Environ. Health Res..

[91] Islam M.S., Mahmud Z.H., Uddin M.H., Islam K., Yunus M., Islam M.S., Nair G.B., Endtz H.P., Sack D.A. (2011). Purification of Household Water using a Novel Mixture Reduces Diarrhoeal Disease in Matlab, Bangladesh. Trans. R. Soc. Trop. Med. Hyg..

[92] Du Preez M., Conroy R.M., Ligondo S., Hennessy J., Elmore-Meegan M., Soita A., McGuigan K.G. (2011). Randomized Intervention Study of Solar Disinfection of Drinking Water in the Prevention of Dysentery in Kenyan Children Aged under 5 Years. Environ. Sci. Technol..

[93] Chiller T.M., Mendoza C.E., Lopez M.B., Alvarez M., Hoekstra R.M., Keswick B.H., Luby S.P. (2006). Reducing Diarrhoea in Guatemalan Children: Randomized Controlled Trial of Flocculant-Disinfectant for Drinking-Water. Bull. World Health Organ..

[94] Clasen T.F., Brown J., Collin S., Suntura O., Cairncross S. (2004). Reducing Diarrhea through the Use of Household-Based Ceramic Water Filters: A Randomized, Controlled Trial in Rural Bolivia. Am. J. Trop. Med. Hyg..

[95] Sima L.C., Desai M.M., McCarty K.M., Elimelech M. (2012). Relationship between Use of Water from Community-Scale Water Treatment Refill Kiosks and Childhood Diarrhea in Jakarta. Am. J. Trop. Med. Hyg..

[96] Conroy R.M., Meegan M.E., Joyce T., McGuigan K., Barnes J. (2001). Solar Disinfection of Drinking Water Protects against Cholera in Children under 6 Years of Age. Arch. Dis. Child..

[97] Rose A., Roy S., Abraham V., Holmgren G., George K., Balraj V., Abraham S., Muliyil J., Joseph A., Kang G. (2006). Solar Disinfection of Water for Diarrhoeal Prevention in Southern India. Arch Dis. Child..

[98] Mäusezahl D., Christen A., Pacheco G.D., Tellez F.A., Iriarte M., Zapata M.E., Cevallos M., Hattendorf J., Cattaneo M.D., Arnold B. (2009). Solar Drinking Water Disinfection (SODIS) to Reduce Childhood Diarrhoea in Rural Bolivia: A Cluster-Randomized, Controlled Trial. PLoS Med..

[99] Freeman M.C., Clasen T., Dreibelbis R., Saboori S., Greene L.E., Brumback B., Muga R., Rheingans R. (2014). The Impact of a School-Based Water Supply and Treatment, Hygiene, and Sanitation Programme on Pupil Diarrhoea: A Cluster-Randomized Trial. Epidemiol. Infect..

[100] Arnold B.F., Colford J.M. (2007). Treating Water with Chlorine at Point-Of-Use to Improve Water Quality and Reduce Child Diarrhea in Developing Countries: A Systematic Review and Meta-Analysis. Am. J. Trop. Med. Hyg..

[101] Du Preez M., Conroy R.M., Wright J.A., Moyo S., Potgieter N., Gundry S.W. (2008). Use of Ceramic Water Filtration in the Prevention of Diarrheal Disease: A Randomized Controlled Trial in Rural South Africa and Zimbabwe. Am. J. Trop. Med. Hyg..

[102] Opryszko M.C., Majeed S.W., Hansen P.M., Myers J.A., Baba D., Thompson R.E., Burnham G. (2010). Water and Hygiene Interventions to Reduce Diarrhoea in Rural Afghanistan: A Randomized Controlled Study. J. Water Health.

[103] Pavlinac P.B., Naulikha J.M., Chaba L., Kimani N., Sangaré L.R., Yuhas K., Singa B.O., John-Stewart G., Walson J.L. (2014). Water Filter Provision and Home-Based Filter Reinforcement Reduce Diarrhea in Kenyan HIV-Infected Adults and their Household Members. Am. J. Trop. Med. Hyg..

[104] Cairncross S., Hunt C., Boisson S., Bostoen K., Curtis V., Fung I.C.H., Schmidt W.P. (2010). Water, Sanitation and Hygiene for the Prevention of Diarrhoea. Int. J. Epidemiol..

[105] Fewtrell L., Kaufmann R.B., Kay D., Enanoria W., Haller L., Colford J.M. (2005). Water, Sanitation, and Hygiene Interventions to Reduce Diarrhoea in Less Developed Countries: A Systematic Review and Meta-Analysis. Lancet Infect. Dis..

[106] Carter M.J. (2005). Enterically Infecting Viruses: Pathogenicity, Transmission and Significance for Food and Waterborne Infection. J. Appl. Microbiol..

[107] Jumaa P.A. (2005). Hand Hygiene: Simple and Complex. Int. J. Infect. Dis..

[108] Kwong L.H., Ercumen A., Pickering A.J., Arsenault J.E., Islam M., Parvez S.M., Unicomb L., Rahman M., Davis J., Luby S.P. (2020). Ingestion of Fecal Bacteria along Multiple Pathways by Young Children in Rural Bangladesh Participating in a Cluster-Randomized Trial of Water, Sanitation, and Hygiene Interventions (WASH Benefits). Environ. Sci. Technol..

[109] Guelinckx I., Tavoularis G., König J., Morin C., Gharbi H., Gandy J. (2016). Contribution of Water from Food and Fluids to Total Water Intake: Analysis of a French and UK Population Surveys. Nutrients.

[110] Kulshreshtha Y., Mota N.J.A., Jagadish K.S., Bredenoord J., Vardon P.J., van Loosdrecht M.C.M., Jonkers H.M. (2020). The Potential and Current Status of Earthen Material for Low-Cost Housing in Rural India. Constr. Build. Mater..

[111] Barreiro C., Albano H., Silva J., Teixeira P. (2013). Role of Flies as Vectors of Foodborne Pathogens in Rural Areas. ISRN Microbiol..

[112] Chan E.Y.Y., Sham T.S.T., Shahzada T.S., Dubois C., Huang Z., Liu S., Hung K.K.C., Tse S.L.A., Kwok K.O., Chung P.-H. (2020). Narrative Review on Health-EDRM Primary Prevention Measures for Vector-Borne Diseases. Int. J. Environ. Res. Public Health.

[113] Chan E.Y.Y., Shahzada T.S., Sham T.S.T., Dubois C., Huang Z., Liu S., Ho J.Y., Hung K.K.C., Kwok K.O., Shaw R. (2020). Narrative Review of Non-Pharmaceutical Behavioural Measures for the Prevention of COVID-19 (SARS-CoV-2) Based on the Health-EDRM Framework. Br. Med. Bull..

[114] (2012). WHO/UNICEF Joint Monitoring Programme for Water Supply and Sanitation. Progress on Drinking Water and Sanitation: 2012 Update. World Health Organization. https://apps.who.int/iris/handle/10665/44842.

[115] Chokshi D.A., Kesselheim A.S. (2008). Rethinking Global Access to Vaccines. BMJ.

[116] Kivuti-Bitok L.W., Chepchirchir A., Waithaka P., Ngune I. (2020). Dry Taps? A Synthesis of Alternative “Wash” Methods in the Absence of Water and Sanitizers in the Prevention of Coronavirus in Low-Resource Settings. J. Prim. Care Community Health.

[117] United Nations ‘Water-Related Diseases Responsible for 80 Per Cent of All Illnesses, Deaths in Developing World’, Says Secretary-General in Environment Day Message, 16 May 2003. https://www.un.org/press/en/2003/sgsm8707.doc.htm.

[118] World Health Organization (2019). Chapter 6-Vaccine Preventable Diseases and Vaccines. International Travel and Health. https://www.who.int/ith/CHAPTER_6_For_Publication.pdf?ua=1.

[119] World Health Organization (2017). Guidelines for Drinking-Water Quality, 4th Edition, Incorporating the 1st Addendum. World Health Organization. https://www.who.int/publications/i/item/9789241549950.

