# Supplementary material for: Narrative Review of Primary Preventive Interventions against Water-Borne Diseases: Scientific Evidence of Health-EDRM in Contexts with Inadequate Safe Drinking Water
Source: Int J Environ Res Public Health. 2021 Nov 23;18(23):12268. doi: 10.3390/ijerph182312268 (PMC8656607; doi:10.3390/ijerph182312268)
Supplement: Supplementary file 1 [file ijerph-18-12268-s001.zip › ijerph-1405057-supplementary.pdf]

Supplementary Materials

Table S1: Relevant interventions, study design, relevant key findings, and conclusion of each utilized reference.

Ref. No.	Title	Journal or Publication (Abbreviated)	Year of Publication	Relevant Intervention(s) (See Table S2)	OCEBM Levels of Evidence Based on Study Design (See Table 1)	Relevant Key Finding(s) and/or Conclusion
[18]	Examining the relationship between community participation and water handling hygiene practices in the informal neighbourhoods of Kisumu, Kenya	Habitat International	April 2017	H	Level 4: Cross-sectional study on community participation on 340 households in informal settlements	• Strong relationship between community participation and improved water handling hygiene practices<br>• Community participation group reported lower incidence of WBD (33% vs 52%), increased tendency to clean water storage (83% vs 17%) and less instances of odour in water
[19]	The impact of water on health and ill-health in a sub-Saharan African wetland: Exploring both sides of the coin	Science of The Total Environment	May 2018	A, E, F	Level 2B: Cohort study of 400 households living around Ewaso Narok Swamp	• Households with improved water source (OR 0.2) and better sanitary hygiene (OR 0.4) were at lower risk of abdominal complaints<br>• Draining stagnant water was also associated with reduced risk of fever (odds ratio of 0.3)<br>• WASH, environmental hygiene and human behaviour have the potential to promote human health in wetlands
[20]	Sanitation and Hygiene-Specific Risk Factors for Moderate-to-Severe Diarrhoea in Young Children in the Global Enteric Multicentre Study, 2007-2011: Case-Control Study	PLoS Medicine	May 2016	A, F	Level 3B: Case-control study identifying risk factors for diarrhoea in young children	• Households with shared sanitation facility had increased risks of moderate to severe diarrhoea (MSD) compared to those with private sanitation facilities (OR 1.4)<br>• Use of soap or ash demonstrated protective effect against MSD in children <5yo (OR 0.7)<br>• More research needed to understand the risks posed by shared facilities in enteric

						disease and to identify cost-effective strategies for remediation
[21]	Assessment of water, sanitation and hygiene interventions in response to an outbreak of typhoid fever in Neno District, Malawi	PLoS One	February 2018	A, C, H	Level 2C: Cohort study of WASH intervention during typhoid outbreak	• Usage of waterguard and soap was low despite distribution, future WASH efforts should be more forceful and sustainable<br>• Knowledge of relationship between drinking unsafe water, poor hygiene and typhoid fever was less than ideal among respondents in community educational group<br>• Low intervention uptake may be due to underlying scepticism about waterborne transmission (ancestral curse, witchcraft)
[22]	The effect of SODIS water treatment intervention at the household level in reducing diarrheal incidence among children under 5 years of age: a cluster randomized controlled trial in Dabat district, northwest Ethiopia	BMC Trials	July 2018	C	Level 1B: Randomised controlled trial examining the effect of SODIS on diarrhoeal incidence	• 40% significant reduction in diarrhoea incidence in children under the age of 5 (U5C)<br>• More effective in 3-5 years old (54% reduction) compared to 2-3 years old (46% reduction)<br>• SODIS has a potential role for diarrhoea prevention inn communities with inadequate safe water due to effectiveness and high adherence rate (91%)
[23]	The Effect of Improved Water Supply on Diarrhea Prevalence of Children under Five in the Volta Region of Ghana: A Cluster-Randomized Controlled Trial	International Journal of Environmental Research and Public Health	September 2015	G	Level 1B: Randomised controlled trial examining the effect of improved water supply on diarrhoeal prevalence	• 11% of reduction in diarrhoea prevalence in children <5yo after improved water source by drilling or rehabilitating boreholes<br>• Intervention effectiveness is also dependent on pre-intervention water quality and hygiene behaviour

[24]	Interventions to improve disposal of human excreta for preventing diarrhoea	Cochrane Library	June 2010	F	Level 1A: Systematic review of RCTs comparing interventions aimed at improving human excreta disposal to prevent diarrhoea	• 11 out of 13 RCTs reported that interventions aimed at improving excreta disposal were protective against diarrhoea<br>• Only 5 out of 13 RCTs focused solely on excreta disposal and the rest combined other sanitation interventions<br>• Ways of excreta disposal improvement mentioned in the review includes latrines, borehole latrines, household flush toilets, private latrines connected to piped water system, private water sealed toilets<br>• Comparability of results were limited due to differences in study populations and settings, in baseline sanitation levels, water and hygiene practices
[25]	Interventions to improve water quality for preventing diarrhoea	Cochrane Library	October 2015	C	Level 1A: Systematic review of randomised controlled trials comparing water quality interventions for diarrhoea prevention	• Distribution of water disinfection products reduced diarrhoea by around a quarter

						(RR 0.77-0.69) but substantial heterogeneity in the size of the effect<br>• Point-of-use filtration reduced diarrhoea by around a half (RR 0.48), which could be used as an important measure until water infrastructure is improved<br>• Important reduction in diarrhoea prevalence with filters (RR 0.39-0.69)<br>• Plumbed in filters were evaluated in high income settings only (RR 0.81)<br>• SODIS in low-income settings reduced diarrhoea by around a third (RR 0.62)<br>• Comparisons between these estimates do not provide evidence of superiority of one intervention over another, as they were confounded by study settings, designs and populations
[26]	A Qualitative Assessment of Beliefs, Attitudes, and Behaviours Related to Diarrhea and Water Filtration in Rural Kenya	American Journal of Public Health	August 2011	C	Level 2C: Outcomes research of water filtration devices distributed as part of a public health campaign	• 35% indicated that they or their families no longer experience stomach problems<br>• Participants regarded filter use as a positive experience: improvements in clean water, decreases in diarrhoeal disease and increased savings (not having to buy additional resources to clean water and other medical expenses)<br>• Some participants offered technical suggestions for device improvements: improving water flow in straws and providing repair for defunctioning devices

[27]	An analysis of the effectiveness of WASH interventions in relation to diarrhoeal diseases in Chipinge district, Zimbabwe	Physics and Chemistry of the Earth	November 2018	G, H	Level 2C: Ecological study to determine existing WASH interventions and assess effectiveness of interventions in a ward of Chipinge district to mitigate diarrhoeal diseases	• WASH interventions such as borehole rehabilitation, latrine construction and hygiene education are in place though they were inadequate<br>• Increase in cases of diarrhoea between 2005-2011 despite various WASH interventions<br>• Positive behavioural change observed with increase in the number of households with hygiene enabling facilities such as rubbish pits and pot racks<br>• Communities had good knowledge on proper hygiene practice, but economic hardships prevented them from practising<br>• There is a need to provide more water supply and sanitation facilities<br>• Health and hygiene education should be intensified by lengthening sessions for sustainability of the interventions
[28]	Effectiveness of a community-based water, sanitation, and hygiene (WASH) intervention in reduction of diarrhoea among under-five children: Evidence from a repeated cross-sectional study (2007-2015) in rural Bangladesh	International Journal of Hygiene and Environmental Health	September 2019	E, F, G, H	Level 4: A cross-sectional study to investigate the effectiveness of the WASH intervention on reduction of diarrhoea in U5C from 2007-2011	• Decreased prevalence of diarrhoea in U5C from 13.7% to 3.6%<br>• Decline in diarrhoea stalled after 2011 and this may be due to lack of improvement in unsafe disposal of faeces and unclean latrine condition after intervention<br>• Necessity of maintaining a small-scale monitoring component involving local community for periodic

						monitoring at household levels for a more sustainable behavioural change<br>• Promotion of safe disposal, maintenance of latrine cleanliness and handwashing are important to ensure reduction in childhood diarrhoea prevalence as merely increasing sanitation coverage may be inadequate
[29]	Comparison of boiling and chlorination on the quality of stored drinking water and childhood diarrhoea in Indonesian households	Epidemiology & Infection	September 2017	C	Level 4: Case series of communities using water treatment methods and diarrhoeal prevalence	• Lower risk of diarrhoea in U5C and E. coli contamination in stored water among households who used Air RahMat (commercial chlorination product) compared to traditional boiling<br>• Low use of Air Rah Mat (7%) despite effectiveness which may be due to poor product acceptability (poor smell, not knowing enough about product, boiling is cheaper and more convenient)<br>• Boiling is highly effective but insufficient heating may not kill all waterborne microbes compared to treated water
[30]	Diarrhoea prevention in a high-risk rural Kenyan population through point-of-use chlorination, safe water storage, sanitation, and rainwater harvesting	Epidemiology & Infection	January 2008	C, D, F, G	Level 2B: Individual cohort study with weekly active diarrhoea surveillance for 8 weeks	• Chlorinating stored water (RR 0.44), latrine presence (RR 0.71), rainwater use (RR 0.70) and living in intervention village (RR 0.31) were independently associated with lower diarrhoea risk<br>• Diarrhoea risk was higher among shallow well users (RR 1.78)<br>• Combined interventions may have increased health impact

[31]	Household Water Treatment and Safe Storage in Low-Income Countries	Encyclopaedia of Environmental Health (Second Edition)	January 2019	C, D	Level 5: Report reviewing the evidence concerning the effectiveness and cost-effectiveness of household water treatment and safe storage to prevent diarrhoeal disease	• Multiple reviews found household treatment and storage to be protective against diarrhoea<br>• Ceramic filters, biosand systems, and LifeStraw filters showed important reductions in diarrhoeal episodes<br>• Source-based interventions had no significant protective effect against diarrhoea
[32]	Effect of hygiene promotion on the risk of reinfection rate of intestinal parasites in children in rural Uzbekistan	Transactions of the Royal Society of Tropical Medicine and Hygiene	June 2007	H	Level 2B: Cohort study assessing the risk of parasitic infection after hygiene promotion	• Risk of reinfection by parasites was 30% lower in hygiene promotion group than treatment group (medicine) and 37% lower than seasonality group<br>• Hygiene promotion can be an effective tool to reduce the risk of reinfection by parasites
[33]	Impact on diarrhoeal illness of a community educational intervention to improve drinking water quality in rural communities in Puerto Rico	BMC Public Health	April 2010	H	Level 2B: Cohort study assessing the burden of diarrhoeal disease that could be prevented by improved management and operation of supplies	• Diarrhoeal illness rates were lower in intervention (2.5%) compared to control communities (3.6%) but this was not statistically significant<br>• Intervention system and owning a dog was negatively associated with diarrhoeal illness<br>• Size of the system and problems with sewage system was positively associated with diarrhoeal illness<br>• Educational interventions directed at identified individuals and community could provide a way of allowing them to manage own drinking water quality; thorough further research with RCT is needed to confirm effectiveness

[34]	Understanding the Challenges of Improving Sanitation and Hygiene Outcomes in a Community Based Intervention: A Cross-Sectional Study in Rural Tanzania	International Journal of Environmental Research and Public Health	June 2017	E, F, G, H	Level 4: Cross-sectional study examining the impact of a four-year intervention to reduce waterborne diseases	• Incidence of diarrhoea among children <5yo increased over intervention period despite improved WASH access<br>• Availability of water alone may not influence the incidence of waterborne diseases – other factors (water storage, safe excreta disposal, hygiene practices) are critical in reducing the spread of WBD
[35]	Evaluation of Student Handwashing Practices During a School-Based Hygiene Program in Rural Western Kenya, 2007	International Quarterly of Community Health Education	January 2017	A, C, G, H	Level 4: Mixed methods approach using quantitative and focus group discussions to evaluate the impact of water quality and hand hygiene programmes on handwashing practices among students	• Fewer incidence of stomach aches and diarrhoea among pupils<br>• Location (<10m from latrine) of water station increases handwashing behaviour with observed improvement in pupils' hygiene reported by teachers<br>• 59% of stations observed to have soap and water and treated drinking water 4 months after implementation
[36]	Comparison of the burden of diarrhoeal illness among individuals with and without household cisterns in northeast Brazil	BMC Infectious Diseases	February 2013	D	Level 4: Cross-sectional study of the prevalence of diarrhoea given the use of cisterns for drinking water	• Significant lower prevalence of diarrhoea (11% vs 18%) in people living in households with cisterns and U5C (16% vs 27%)<br>• No significant differences found in terms of types of symptoms and duration of illness
[37]	Community-based interventions for preventing diarrhoea in people living with HIV in sub-Sahara Africa: A systematic review	Malawi Medical Journal	March 2019	C, D	Level 2A: Systematic review of cohort studies examining community-based interventions for diarrhoea prevention in people living with HIV	• Community-based interventions such as water treatment and safe storage were associated with 20-53% reduction in diarrhoea among person living with HIV (PLHIV)<br>• Largest reduction rate resulted from using One Life Straw Family filter and two 5L safe storage containers to prevent water contamination

[38]	Use, adoption, and effectiveness of tippy-tap handwashing station in promoting hand hygiene practices in resource-limited settings: a systematic review	BMC Public Health	June 2020	G	Level 5: Systematic review using different types of studies to assess the use, benefits, adoption and effectiveness of tippy-tap handwashing stations	• Availability of tippy-taps increased handwashing and soap use<br>• Majority of participants who were oriented to tippy-taps or recruited to the studies built their tippy-tap stations after promotional activities have ended<br>• Only one study measured diarrhoeal rates as an outcome and tippy-taps were reported to be effective in preventing stomach pain among participants; further studies required to evaluate effectiveness on preventing WBD
[39]	Are hygiene and public health interventions likely to improve outcomes for Australian Aboriginal children living in remote communities? A systematic review of the literature	BMC Public Health	May 2008	A, E, F, G, H	Level 1A: Systematic review of randomised controlled trials assessing the relationship between interventions and infectious diseases	• Clear evidence in the effect of education and handwashing with soap in preventing diarrhoeal disease among children<br>• Some evidence of an effect of education and other hygiene behaviour change intervention, but the size of these effects is small, and quality is generally poor<br>• Multifaceted interventions are likely to provide greatest opportunity to improve child health outcomes
[40]	Dramatic Reduction in Diarrhoeal Diseases through Implementation of Cost-Effective Household Drinking Water Treatment Systems in Makwane Village, Limpopo Province, South Africa	International Journal of Environmental Research and Public Health	February 2018	C	Level 2C: Outcomes research on the cost-effectiveness of household water treatment systems for the reduction of diarrhoeal diseases	• Both filters reduced the incidence of diarrhoea by 96.2% in intervention group compared to control<br>• Most episodes of diarrhoea were reported by U5C, followed by group aged >56 yo<br>• Major reduction in diarrhoea in U5C (90.5%)<br>• Demonstrates importance of household water treatment systems in

						rural areas to reduce diarrhoeal diseases
[41]	Assessing water filtration and safe storage in households with young children of HIV-positive mothers: a randomized, controlled trial in Zambia	PLoS One	October 2012	C, D	Level 1B: Randomised controlled trial assessing water filters and jerry cans in reducing diarrhoea	• 96% of households reported filter use<br>• Associated with reductions in longitudinal prevalence of reported diarrhoea: 53% reduction in children <2yo and 54% reduction in other household members<br>• Quality of water stored was significantly better in intervention households<br>• Correct use of water filter combined with safe storage is protective against diarrhoea in HIV population
[42]	Evidence on the Effectiveness of Water, Sanitation, and Hygiene (WASH) Interventions on Health Outcomes in Humanitarian Crises: A Systematic Review	PLoS One	September 2015	A, C, D	Level 5: Systemic review on quantitative studies including both randomised and non-randomised trials to examine the quantity and quality of evidence on WASH interventions	• Studies that employed point of use water quality intervention showed a statistically significant change in health outcome: 50% using safe water storage and 35% using household water treatment<br>• Impact of WASH interventions on health outcomes in humanitarian crises remain extremely limited
[43]	Keeping clean water clean in a Malawi refugee camp: a randomized intervention trial	Bulletin of the World Health Organization	July 2001	D	Level 1B: Individual RCT to assess the ability of a water container with a cover and a spout to prevent household contamination of water	• 69% reduction in geometric mean of faecal coliform levels in household water<br>• 31% less diarrhoea incidence in U5C<br>• Visible faeces in latrine and presence of animals significantly associated with increased diarrhoea in children<br>• Bucket use is more popular in camp inhabitants compared to proper chlorination (although less expensive and more effective) and this may be due to culturally appropriate design of bucket

[44]	Effect of household water treatment with chlorine on diarrhoea among children under the age of five years in rural areas of Dire Dawa, eastern Ethiopia: a cluster randomized controlled trial	Infectious Diseases of Poverty	June 2020	C	Level 1B: Randomised controlled trial evaluating the effectiveness of water treatment on childhood diarrhoea	• 36% reduction in diarrhoea rate: highest reduction in 1-2 yo (42.7%) and lowest reduction in 3-4 yo (30.4%)<br>• High adherence to intervention (81.3%) as measured by free residual chlorine test<br>• Chlorinating drinking water at household level may be a valuable interim solution for reducing incidence of diarrhoea until portable water is made accessible
[45]	Integrating Household Water Treatment, Hand Washing, and Insecticide-Treated Bed Nets into Paediatric HIV Care in Mombasa, Kenya: Impact on Diarrhea and Malaria Risk	Journal of Acquired Immune Deficiency Syndromes	November 2017	A, C, D	Level 4: Retrospective case series of the association between the Healthy Living Initiative and the risk of diarrhoea and malaria among HIV-infected children	• HIV-infected children in healthy livening initiative had 71% lower risk of diarrhoea and 87% lower risk of malaria<br>• No independent association between antiretroviral use and opportunistic infections in HIV infected children
[46]	The impact of hygiene and localised treatment on the quality of drinking water in Masaka, Rwanda	Journal of Environmental Science and Health Part A – Toxic/Hazardous Substances & Environmental Engineering	March 2014	A, C	Level 4: Cross-sectional study to evaluate the socio-demographic factors, water quality, water handling and sanitation practices in a rural Rwandan community	• Despite significant reduction in coliforms and faecal coliforms following treatment, water at point of use was found unsafe<br>• Higher frequency of diarrhoeal diseases among people who did not wash hands before food preparation and after toilet<br>• Statistically significant association between education levels and water treatment practices at the households<br>• Participants had limited knowledge regarding water storage practice for prevention of water contamination<br>• Education is a fundamental precursor to advocate water treatment at point of use

[47]	Association of nutrition, water, sanitation and hygiene practices with children's nutritional status, intestinal parasitic infections and diarrhoea in rural Nepal: a cross-sectional study	BioMed Central Public Health Journal	Aug 2020	A, C, E, F	Level 4: cross-sectional study of 1427 households to assess the association of hygiene practice with parasitic infections and diarrhoea	• Children from households experiencing interruption of water supply had 2.87 higher odds of diarrhoea than those without interruption<br>• Children of caregivers who were aware of handwashing needs were better protected against diarrhoea<br>• Children with clean latrines were significantly better protected<br>• Children with visually clean hands were significantly better protected against diarrhoea than those with dirty hands<br>• Children living in households with mud floor had 2.29 times higher odds of suffering from diarrhoea than cement floor
[48]	Diarrhoea prevalence in a randomized, controlled prospective trial of point-of-use water filters in homes and schools in the Dominican Republic	Journal of Tropical Medicine and Health	Jan 2021	C	Level 1B: Randomised controlled trial examining the effect of intervention strategies on water transport and storage on counts of E. coli	• Decrease in diarrhoea prevalence from 25.6 to 9.76% from installation to follow up (7-200 days post installation)<br>• Declines were also observed in diarrhoea with economic or educational consequences (medical treatment of days of missing school and work) from 9.64% to 1.57%
[49]	Seasonal prevalence, risk factors, and One Health intervention for prevention of intestinal parasitic infection in underprivileged communities on the Thai communities on the Thai Myanmar border	Internal Journal of Infectious Diseases	April 2021	H	Level 2B: Cohort study evaluating the effectiveness of One Health intervention for prevention of intestinal parasitic infection in Thai communities	• 22.6% reduction in symptomatic cases of intestinal parasitic infections<br>• Most significant reduction was found in E. histolytica like-amoeba (2.9%), followed by A. lumbricoides (2%)<br>• 51.5% of respondents had no knowledge of causes of diarrhoea and

						71.3% had basic understanding after intervention<br>• 32.3% had no treatment of drinking water before intervention but all respondents treated water after health advocacy
**Additional papers identified through snowballing method**						
[50]	A cluster randomized controlled trial to reduce childhood diarrhoea using hollow fibre water filter and/or hygiene-sanitation educational interventions	The American Journal of Tropical Medicine and Hygiene	July 2014	C, H	Level 1B: Randomized controlled trial evaluating the efficacy of both interventions to reduce diarrhoeal disease in children under 5	• Significant reduction in diarrhoea disease among U5C in filter (85%) and WASH + filter (78%) arms compared to control<br>• Non-significant reduction in diarrhoeal prevalence in WASH arm<br>• Distribution of filter alone may be sufficient to reduce diarrhoea outcomes
[51]	A randomized controlled trial of household-based flocculant-disinfectant drinking water treatment for diarrhoea prevention in rural Guatemala	The American Journal of Tropical Medicine and Hygiene	October 2003	C	Level 1B: Randomized controlled trial of drinking water treatment to prevent diarrhoea in Guatemala	• All water treatment intervention reported decrease in incidence of diarrhoea<br>• Intervention with flocculant-disinfect and vessels had the largest decrease in the incidence of diarrhoea (29%) and highest concentration of free chlorine (35%)<br>• Intermittent use of home water treatment, where diarrhoea was a leading cause of death, could reduce incidence of diarrhoea
[52]	A randomized controlled trial of the concrete Biosand filter and its impact on diarrheal disease in Bonao, Dominican Republic	The American Journal of Tropical Medicine and Hygiene	February 2009	C	Level 1B: Randomized controlled trial of the effectiveness of the biosand filter on diarrhoea	• Households using the filter experienced a significant protective effect against waterborne diarrhoeal disease (0.53 OR)<br>• Households using the filter had a significantly improved drinking water

						quality compared to the control households
[53]	A randomized controlled trial of the plastic-housing BioSand filter and its impact on diarrheal disease in Copan, Honduras	The American Journal of Tropical Medicine and Hygiene	June 2012	C	Level 1B: Randomized controlled trial assessing the impact of the plastic-housing BioSand filter on diarrhoeal disease	• 45% reduction in the Incidence of diarrhoea in U5C in households with filter use<br>• Reduction fluctuated seasonally and was found not to be statistically significant<br>• Household with filter reported significantly better drinking water quality regardless of water source or season
[54]	A Stepped Wedge Cluster-Randomized Trial Assessing the Impact of a Riverbank Filtration Intervention to Improve Access to Safe Water on Health in Rural India	The American Journal of Tropical Medicine and Hygiene	March 2020	G	Level 1B: Cluster-randomized trial assessing whether riverbank filtration-treated water reduced diarrhoea	• No measurable reduction in diarrhoea post-intervention and this could be due to low intervention uptake, availability of other water resources, and low baseline diarrhoeal prevalence<br>• Study highlights the difficulties in evaluating the impact of real-world interventions and the potential for an optimized RCT design to address constraints in such evaluation
[55]	A stepped wedge, cluster-randomized trial of a household UV-disinfection and safe storage drinking water intervention in rural Baja California Sur, Mexico	The American Journal of Tropical Medicine and Hygiene	August 2013	C, D	Level 1B: Cluster-randomized trial assessing the impact of UV disinfection and safe storage of water on diarrhoea prevalence	• Increase in the percentage of households with access to treated and safely stored drinking water (23-62%)<br>• Reduction in the percentage of households with E. coli contaminated drinking water<br>• No significant reduction in diarrhoea was observed so future studies would require a larger study population

						with higher baseline diarrhoea prevalence
[56]	A study to evaluate the acceptability, feasibility and impact of the packaged interventions ("Diarrhea Pack") for prevention and treatment of childhood diarrhoea in rural Pakistan	BMC Public Health	October 2013	B, C, H	Level 1B: Cluster-randomized trial evaluating the effectiveness of the "Diarrhea Pack" on diarrhoea	• Significant increase in uptake of ORS and Zinc along with reduction in antibiotics use, diarrhoea burden and hospitalization<br>• Diarrhoea pack was well-accepted within the community and showed feasibility for delivery through community. It has the potential to be scaled up to a national level
[57]	A systematic review of the health outcomes related to household water quality in developing countries	Journal of Water and Health	March 2004	C, D	Level 2A: Systematic review of observational studies investigating relationship between water quality and diarrhoea/cholera, and water treatment and storage interventions	• Clear relationship was found in cholera with contaminated water<br>• No clear relationship between general diarrhoea and water quality but interventions did significantly reduce diarrhoeal incidence<br>• Water policy could be implemented to reduce diarrhoeal morbidity
[58]	Assessing the impact of drinking water and sanitation on diarrhoeal disease in low- and middle-income settings: Systematic review and meta-regression	Tropical Medicine & International Health	August 2014	C, D, E, F	Level 1A: Systematic review of randomized controlled trials and observational studies assessing the impact of water and sanitation improvements on diarrhoeal disease	• Improvements in intervention studies focused on drinking water and sanitation were both associated with decreased risks of diarrhoea, but notable difference illness reduction according to type<br>• Specific improvements (water filter use, provision of high-quality piped water, sewer connections) were associated with greater reductions in diarrhoea

[59]	Chlorination and safe storage of household drinking water in developing countries to reduce waterborne disease	Water Science & Technology	February 2003	C, D	Level 1B: Randomized controlled trial evaluating the impact of chlorination and safe water storage to reduce diarrhoea	• E. coli in stored waters were <1/100mL in most intervention households but readily detectable at high levels in control<br>• Intervention households also lower levels of C. perfringens and heterotrophic plate count bacteria compared to control<br>• 43% reduction in community diarrhoea in Bolivia (1.25 monthly episodes in intervention group vs 2.2 in control)<br>• 24% reduction in child diarrhoea in Bangladesh (19.6 vs 24.8 mean episodes)<br>• Simple treatment with chlorine and storage system for non-piped domestic water has potential to reduce global burden of waterborne diarrhoeal disease
[60]	Combining drinking water treatment and hand washing for diarrhoea prevention, a cluster randomised controlled trial	Tropical Medicine & International Health	April 2006	A, C	Level 1B: Randomized controlled trial assessing the impact of water treatment and handwashing to prevent diarrhoea in Pakistan	• Intervention group had lower prevalence of diarrhoea; higher reduction in disinfectant (64%) compared to bleach (55%)<br>• No benefit in reduction of diarrhoea incidence when water treatment intervention is combined with handwashing promotion (55% and 51%)
[61]	Delayed effectiveness of home-based interventions in reducing childhood diarrhoea, Karachi, Pakistan	The American Journal of Tropical Medicine and Hygiene	October 2004	A, C, D	Level 2B: Cohort study evaluating the effectiveness of home-based interventions to reduce childhood diarrhoea in Karachi squatter settlements	• In 2000: 73% reduction in diarrhoea in children in water treatment intervention with 56% reduction in those who received soap; no reduction from either intervention in households without a refrigerator

						• In 2001, the interventions were equally effective with or without a refrigerator<br>• Wealthy households are able to adapt behavioural change more quickly
[62]	Diarrhea prevention in a Kenyan school through the use of a simple safe water and hygiene intervention	The American Journal of Tropical Medicine and Hygiene	February 2007	C, D, E	Level 2C: Outcomes research of a pilot project in a Kenyan school to prevent diarrhoea	• Project implementation reduced clinic visits for diarrhoea from 130 (at 2000) to<br>71 (at 2003) and to o 13 (at 2004)<br>• This saved the school $5.49 per student per year and has been expanded to 70 schools with a planned evaluation
[63]	Diarrhea prevention in people living with HIV: an evaluation of a point-of-use water quality intervention in Lagos, Nigeria	AIDS Care	March 2011	C, D	Level 4: A case series study on water interventions and diarrhoea prevention in people living with HIV	• 36% lower diarrhoeal rates in post-intervention period<br>• In participants with residual chlorine in stored water in >85% of home visits, there was a 46% reduction rate in diarrhoea<br>• In participants with residual chlorine in stored water in <85% of home visits, there was no significance in diarrhoea reduction<br>• Point-of-use treatment was associated with reduced diarrhoea risk in PLHIV, but regular water treatment was required to achieve such health benefits
[64]	Diarrhea prevention through household-level water disinfection and safe storage in Zambia	The American Journal of Tropical Medicine and Hygiene	May 2002	C, D, H	Level 1B: Randomized controlled trial comparing the efficacy of water disinfection and safe storage at diarrhoea prevention compared to control households	• High compliance: 97% households reported using disinfectant and 72-95% with measurable chlorine<br>• Stored water in intervention households was significantly less contaminated with E. Coli than water in control households

						• 48% lower diarrhoeal disease risk for individuals in intervention households<br>• Intervention is useful for preventing WBD in families in developing countries who lack access to potable water
[65]	Effect of a point-of-use water treatment and safe water storage intervention on diarrhoea in infants of HIV-infected mothers	The Journal of Infectious Diseases	October 2009	C, D, H	Level 2B: Cohort study of infants of HIV-infected mothers and diarrhoea prevalence	• Infants who underwent intervention had less diarrhoea before and after weaning but<br>• No differences in the frequency of diarrhoea between intervention and control during weaning period<br>• High compliance indicated by high range of chlorination in testing water (80-95%)<br>• Provision of safe water may be insufficient to prevent weaning-associated diarrhoea
[66]	Effect of chlorination of drinking-water on water quality and childhood diarrhoea in a village in Pakistan	Journal of Health, Population and Nutrition	March 2003	C	Level 2B: Cohort study assessing the impact of chlorinating the public-water supply system in a Pakistan village and its effects on childhood diarrhoea	• Higher risk of diarrhoea in children using chlorinated water compared to those using groundwater source<br>• Incidence of diarrhoea in the intervention village was not statistically different from those in a neighbouring village where most children used non-chlorinated water<br>• In this study, chlorinating drinking water does not seem to be a priority intervention to reduce childhood diarrhoea
[67]	Effect of city-wide sanitation programme on reduction in rate of childhood diarrhoea in northeast Brazil: assessment by two cohort studies	The Lancet	November 2007	G	Level 2B: Longitudinal study of a city-wide sanitation intervention to improve sewerage coverage in Brazil	• 21% reduction in prevalence of diarrhoea post-intervention<br>• Urban sanitation (increase access to adequate sewer system) is a highly effective health measure

[68]	Effect of handwashing on child health: a randomised controlled trial	The Lancet	July 2005	A	Level 1B: Randomized controlled trial assessing the impact of promoting handwashing with soap on the incidence of diarrhoea and other common childhood diseases	• 53% reduction in diarrhoea incidence in children <15yo in households with soap<br>• Accompanied by 50% lower incidence of pneumonia in U5C and 34% lower incidence of impetigo in children <15yo<br>• Incidence of disease was not significantly different in households with plain soap or antibacterial soap<br>• Handwashing with soap prevents two clinical syndromes that cause the largest number of childhood deaths globally
[69]	Effect of home-based water chlorination and safe storage on diarrhoea among persons with human immunodeficiency virus in Uganda	The American Journal of Tropical Medicine and Hygiene	November 2005	C, D, H	Level 1B: Randomized controlled trial of a home-based safe water intervention on the incidence of diarrhoea in people living with HIV in Uganda	• 25% reduction of diarrhoeal episodes, 33% fewer days with diarrhoea and less visible blood or mucus in stool<br>• Equally effective in those with or without prophylaxis treatment: reduced diarrhoea episodes by 67% in total<br>• Safe water system reduced diarrhoea frequency and severity PLHIV in Uganda
[70]	Effect of household-based drinking water chlorination on diarrhoea among children under five in Orissa, India: a double-blind randomised placebo-controlled trial	PLoS Medicine	August 2013	C	Level 1B: Randomized controlled trial of chlorination and the impact on childhood diarrhoea in India	• Longitudinal prevalence of diarrhoea among intervention and control children remained the same: no evidence of protectiveness<br>• Low compliance may have contributed to lack off effect as only 32% of water sample tested positive for residual chlorine<br>• Modest reduction in water contamination (50 vs 122 per 100ml)
[71]	Effect of in-line drinking water chlorination at the point of collection on child diarrhoea in urban	The Lancet	September 2019	C	Level 1B: Randomized controlled trial assessing the effect of automatic water chlorination at the point of collection	• Children in treatment group had less diarrhoea than control group (RR of 0.77)

	Bangladesh: a double-blind, cluster-randomised controlled trial				compared to a control group and its impact on childhood diarrhoea	• Treatment taps had detectable free chlorine residual 83% of the time compared to 0% at control taps<br>• Passive chlorination at point of collection could be an effective and scalable strategy in low-income urban settings
[72]	Effect of intensive hand washing promotion on childhood diarrhea in high-risk communities in Pakistan	The Journal of the American Medical Association	June 2004	A	Level 1B: Randomized controlled trial of low-income neighbourhoods and the impact of promoting handwashing with soap on childhood diarrhoea	• 53% lower incidence of diarrhoea, 39% fewer days with diarrhoea in <15yo<br>• 42% fewer days with diarrhoea in severely malnourished children<br>• Similar reductions in diarrhoea among children with antibacterial soap<br>• Improvement in handwashing is effective in reducing diarrhoea among children at high risk of death of diarrhoea
[73]	Effect of washing hands with soap on diarrhoea risk in the community: a systematic review	The Lancet Infectious Diseases	May 2003	A	Level 1C: Systematic review of intervention, case-control, cross-sectional, and cohort studies assessing the impact of handwashing with soap on the risk of diarrhoea	• Handwashing could reduce diarrhoea risk by 47% from pooled relative risk<br>• Handwashing with soap could reduce diarrhoea risk by 42-44%<br>• 48% reduction in intestinal infections and 59% reduction in shigellosis<br>• Better-designed trials are needed to measure impact of handwashing as many of the studies had poor quality and may be inflated by publication bias
[74]	Effects of a large-scale distribution of water filters and natural draft rocket-style cookstoves on diarrhoea and acute respiratory infection: A cluster-randomized controlled	PLoS Medicine	June 2019	C	Level 1B: Randomized controlled trial assessing the impact of water filters and cookstoves on diarrhoea in children under 5 in a cluster in Rwanda	• Intervention reduced childhood diarrhoea prevalence by 29% and childhood acute respiratory infection by 25%<br>• Intervention reduced the number of households with detectable faecal

	trial in Western Province, Rwanda					contamination in drinking water by 38%<br>• Use of filter and intervention stove decreased throughout follow up<br>• Further research is necessary to determine long-term intervention use and benefits
[75]	Evaluating the sustained health impact of household chlorination of drinking water in rural Haiti	The American Journal of Tropical Medicine and Hygiene	November 2012	C	Level 4: Case series assessing the impact of a safe water programme on diarrhoea disease in Haiti	• Significantly fewer U5C in intervention households had an episode of diarrhoea in the previous 48 hours (OR = 0.41)<br>• Reduction in this long-term program was comparable to those seen in short-term randomized: household chlorination can be an effective long-term strategy
[76]	Evaluation of a pre-existing, 3-year household water treatment and handwashing intervention in rural Guatemala	International Journal of Epidemiology	December 2009	A, C	Level 2B: Cohort study of 30 villages in Guatemala assessing the impact of water treatment and handwashing campaigns	• Some improvements found in water treatment behaviour<br>• No difference in self-reported handwashing behaviour, spot-check hygiene conditions or prevalence of child diarrhoea<br>• Lack of child health impacts is consistent with un-sustained behaviour adoption (post-intervention follow up) which highlights the difficulty in implementing water treatment and handwashing outside intensive trials
[77]	Evaluation of a water, sanitation, and hygiene education intervention on diarrhoea in northern Pakistan	Bulletin of the World Health Organization	May 2003	E, G, H	Level 3B: Case-control study evaluating diarrhoea prevalence in children living in WASEP communities compared to those not living in WASEP communities	• 25% reduction in incidence of diarrhoea in intervention group<br>• Younger children, girls and children of younger mothers were associated with a higher likelihood of diarrhoea
[78]	Hand hygiene intervention strategies to reduce diarrhoea	International Journal of	April 2017	A	Level 1A: Systematic review of randomized controlled trials assessing the impact of	• Hand hygiene can reduce incidence of diarrhoea and respiratory conditions

	and respiratory infections among schoolchildren in developing countries: a systematic review	Environmental Research and Public Health			hand hygiene interventions at reducing diarrhoea	• Three main strategies (training, funding and policy) are often used in combination and known as multi-level hand-washing interventions<br>• Further evidence-based studies with improved methodological rigour to inform policy in the area
[79]	Hand washing for preventing diarrhoea	Cochrane Reviews	January 2008	A, H	Level 1A: Systematic review of randomized controlled trials assessing the impact of hand washing on episodes of diarrhoea	• 29% reduction in diarrhoea episodes in high income countries<br>• 31% reduction in diarrhoea episodes in middle or low-income countries<br>• Effect of intervention on hand hygiene related behavioural outcome in all settings showed an increase<br>• Lack of trials evaluating effects of handwashing interventions on diarrhoea related deaths, all-under-five cause of mortality or costs
[80]	Hand washing with soap and WASH educational intervention reduces under-five childhood diarrhoea incidence in Jigjiga District, eastern Ethiopia: a community-based cluster randomized controlled trial	Preventive Medicine Reports	April 2017	A, H	Level 1B: Randomized controlled trial evaluating the effect of handwashing with soap and WASH educational interventions on diarrhoea in children under 5	• Overall diarrhoeal diseases reduction of 35% in <U5C whose primary caretakers received intervention<br>• Similar effects observed in other WASH educational intervention and could be effective in rural pastoralist area
[81]	Health gains from solar water disinfection (SODIS): evaluation of a water quality intervention in Yaoundé, Cameroon	Journal of Water and Health	December 2010	C	Level 4: Cross-sectional study evaluating the impact of solar disinfection in slum areas in Yaoundé, Cameroon	• Reduction in diarrhoea prevalence from 34.3% to 22.8% in intervention group<br>• intervention experienced an even lesser diarrhoea prevalence at 18.3%<br>• Adoption of intervention was associated with marital status<br>• Effectiveness is observed when other risk factors are considered so further promotional activities in low-income settings are recommended

[82]	High compliance randomized controlled field trial of solar disinfection of drinking water and its impact on childhood diarrhea in rural Cambodia	Environmental Science & Technology	September 2011	C	Level 1B: Randomized controlled trial investigating the effect of solar disinfection of drinking water on the incidence of childhood diarrhoea in Cambodia	• Reduced incidence of dysentery (IRR 0.5) and non-dysentery diarrhoea (IRR 0.37) in intervention group<br>• High study compliance (>90%)<br>• Effective and culturally acceptable point-of use water treatment method that may be of benefit among similar communities in neighbouring South East Asian countries
[83]	Household based treatment of drinking water with flocculant-disinfectant for preventing diarrhoea in areas with turbid source water in rural western Kenya: cluster randomised controlled trial	The British Medical Journal	September 2005	C	Level 1B: Randomized controlled trial comparing the effect of different water treatment on diarrhoea prevalence	• In children <2yo, compared to the controls, there was a 25% reduction of diarrhoea prevalence in flocculant-disinfectant arm, and 17% reduction in chlorination arm<br>• In all age groups, there was a 19% and 26% reduction in diarrhoea with flocculant-disinfectant and chlorination respectively<br>• Fewer deaths reported in both intervention groups compared to controls<br>• Flocculant-disinfectant is suitable for areas with highly contaminated and turbid water in reducing diarrhoea
[84]	Household water chlorination reduces incidence of diarrhea among under-five children in rural Ethiopia: a cluster randomised controlled trial	PLoS One	October 2013	C	Level 1B: Randomized controlled trial to assess the effectiveness of household water chlorination at reducing diarrhoea incidence in children under 5	• Size of diarrhoea reduction varies with different age groups: 63% in 3-4yo, 53% in 1-2yo and 44% in <1yo. This may be due to higher susceptibility and increased chance of exposure to contaminated water

						• Statistically significant reduction in diarrhoea observed in intervention group<br>• Chlorination can help reduce childhood diarrhoea morbidity until reliable access to safe water is achieved
[85]	Household-based ceramic water filters for the prevention of diarrhea: a randomized, controlled trial of a pilot program in Colombia	The American Journal of Tropical Medicine and Hygiene	October 2005	C	Level 1B: Randomized controlled trial exploring the use of ceramic water filters and its effect on diarrhoea prevalence	• 60% lower diarrhoea prevalence in households using filters<br>• More intervention households with no detectable thermotolerant coliforms in water (47.7% vs 0.9%), where 24.2 % conformed to WHO's limits for low risk<br>• However, protective effect and microbiologic performance is not consistent across communities. This suggests a greater need to consider the particular setting before intervention implementation
[86]	Intermittent slow sand filtration for preventing diarrhoea among children in households using unimproved water sources: A randomized controlled trial	Tropical Medicine and International Health	October 2009	C	Level 1B: Randomized controlled trial assessing the impact of the concrete BioSand filter on childhood diarrhoea	• Intervention households had better drinking water quality with significantly fewer diarrhoea days among children up to 15yo (86 days vs 203 days)<br>• BioSand filter produced similar effects as commercial point-of-use products, adding to the range of effective options for water treatment in low-income populations
[87]	Interventions to improve water quality for preventing diarrhoea: systematic review and meta-analysis	The British Medical Journal	April 2007	C, D, E	Level 1A: Systematic review of randomized and quasi-randomised controlled trials of interventions to improve drinking water quality to prevent diarrhoea in settings with endemic disease	• Interventions that aim to improve microbial quality of drinking water are effective in preventing diarrhoea<br>• Effectiveness was not conditioned on the presence of improved water supplies or sanitation

						• Effectiveness was not enhanced by combining the intervention with instructions on basic hygiene, vessel, improved sanitation or environmental interventions to prevent diarrhoea
[88]	Local drinking water filters reduce diarrheal disease in Cambodia: a randomized, controlled trial of the ceramic water purifier	The American Journal of Tropical Medicine and Hygiene	September 2008	C	Level 1B: Randomized controlled trial comparing two household drinking water filters on diarrhoea in a village in Cambodia	• Households using ceramic filter (OR 0.51) and iron ceramic filter (OR 0.58) both reported significantly less diarrhoea<br>• This effect was observed in all age groups and both sexes<br>• Longer-term and larger studies are needed to assess sustainability and reliability of these interventions
[89]	Point-of-use water treatment and diarrhoea reduction in the emergency context: an effectiveness trial in Liberia	Tropical Medicine and International Health	October 2006	C, D	Level 1B: Randomized controlled trial to determine the effectiveness of point-of-use water treatment, and improved water storage on diarrhoea prevalence	• Intervention households with flocculant-disinfectant and improved storage experienced a 90% reduction in diarrhoea incidence and 83% reduction in diarrhoea prevalence compared to the control group with just improved water storage alone<br>• Residual chlorine levels in intervention groups met Sphere standards and had a 95% compliance rate
[90]	Preventing diarrhoea with household ceramic water filters: assessment of a pilot project in Bolivia	International Journal of Environmental Health Research	June 2006	C	Level 1B: Randomized controlled trial assessing the effects of ceramic water filters on diarrhoea prevention in a Bolivian community	• Filter use is associated with a 45.3% reduction in diarrhoeal prevalence<br>• Water filters eliminated thermotolerant coliforms and significantly reduced turbidity (improved water aesthetics)<br>• Modest compliance post-intervention: 67% of regular filter use, 13% of intermittent use and 21% not in use
[91]	Purification of household water using a novel mixture	Transactions of the Royal Society of Tropical	June 2011	C	Level 2B: Cohort study determining the acceptability and effectiveness of a surface water purifying mixture to prevent	• 83 patients were treated for diarrhoea at a local hospital from the control

	reduces diarrhoea disease in Matlab, Bangladesh	Medicine and Hygiene			diarrhoea in a rural community in Bangladesh	group, compared to only one patient from the intervention group<br>• 73 intervention families decided to switch from tube well water to the surface water plus mixture<br>• Mixture could be used as a cheaper, more convenient point-of-use water treatment intervention
[92]	Randomized intervention study of solar disinfection of drinking water in the prevention of dysentery in Kenyan children aged under 5 years	Environmental Science & Technology	November 2011	C	Level 1B: Randomized controlled trial assessing the impact of solar disinfection for the prevention of dysentery in Kenya	• Significant reduction in episodes of dysentery (IRR = 0.56) and non-dysentery diarrhoea (IRR = 0.73)<br>• Significant increase in median height-for-age with 0.23kg difference in weight in intervention<br>• No difference in microbial quality but E. coli concentrations were significantly lower in intervention group
[93]	Reducing diarrhoea in Guatemalan children: a randomized controlled trial of a flocculant-disinfectant for drinking water	Bulletin of the World Health Organization	January 2006	C	Level 1B: Randomized controlled trial of Guatemalan households and the use of a flocculant-disinfectant on the prevalence of diarrhoea	• 40% lower prevalence of diarrhoea compared to those with standard water handling practices<br>• Children <1 yo had 39% lower prevalence of diarrhoea<br>• Successful introduction and long-term use of disinfectant can contribute to reduction of diarrhoea morbidity and mortality
[94]	Reducing diarrhoea through the use of household-based ceramic water filters: a randomized, controlled trial in rural Bolivia	The American Journal of Tropical Medicine and Hygiene	June 2004	C	Level 1B: Randomized controlled trial assessing the impact of ceramic water filters at reducing diarrhoea in Bolivia	• 70% lower diarrhoea disease rate in intervention group<br>• 83% reduction in diarrhoea risk for <5yo<br>• All intervention samples were free from thermotolerant coliforms (vs 15.5%)<br>• Affordable ceramic water filters enable low-income households to

						treat and maintain microbiologic quality of water
[95]	Relationship between use of water from community-scale water treatment refill kiosks and childhood diarrhea in Jakarta	The American Journal of Tropical Medicine and Hygiene	December 2012	C, G	Level 2B: Cohort study investigating the association between diarrhoea risk and community-scale water treatment and refill kiosk	• Significant reduction in rate of diarrhoea in water kiosk group (3.97), bottled water group (3.6), compared to tap water (8.13)<br>• Significant lower diarrhoea risk (RR 0.49) among water kiosk and bottled water users; no difference in risk in well water users<br>• Low-cost water kiosk could be a cost-effective alternative in urban areas with unsafe tap water as there is a similar reduction in diarrhoea risk
[96]	Solar disinfection of drinking water protects against cholera in children under 6 years of age	Archives of Disease in Childhood	October 2001	C	Level 1B: Randomized controlled trial evaluating the effects of solar disinfection on cholera prevalence in children under 6 in a Maasai community	• No significant difference found in the risk of cholera in adults with solar disinfection<br>• In children <6yo, only 3 cases of cholera were reported out of 155 in the intervention group compared to 20 out of 144 in the control group<br>• Results support the effectiveness of solar disinfection in reducing waterborne disease risk in children
[97]	Solar disinfection of water for diarrhoeal prevention in Southern India	Archives of Disease in Childhood	February 2006	C	Level 1B: Randomized controlled trial evaluating the efficacy of solar disinfection on the prevention of diarrhoea in an urban slum in Vellore, Tamil Nadu	• Significant reduction found in the incidence (1.7 vs 2.7 per child-year), duration, and severity of diarrhoea in children drinking solar disinfected water<br>• Risk of diarrhoea was reduced by 40% in those using solar disinfection<br>• Solar disinfection is a suitable method to increase water safety in a resource

						limited environment and significantly reduce diarrhoea morbidity
[98]	Solar drinking water disinfection (SODIS) to reduce childhood diarrhoea in rural Bolivia: a cluster-randomized, controlled trial	PLoS Medicine	August 2009	C	Level 1B: Randomized controlled trial evaluating the effect of solar disinfection on reducing diarrhoea in children under 5	• Incidence rate of diarrhoea in the intervention group was 3.6 compared to 4.3 episodes/year in the control group<br>• Median length of diarrhoea was the same in both groups (3 days)<br>• Compliance was only moderate and there was not a substantial reduction in diarrhoea reported in children<br>• Need for better evidence of how the well-established laboratory efficacy translates into field effectiveness
[99]	The impact of a school-based water supply and treatment, hygiene, and sanitation programme on pupil diarrhoea: a cluster-randomized trial	Epidemiology & Infection	February 2014	C, G, H	Level 1B: Randomized controlled trial assessing the impact of school-based WASH interventions on diarrhoea in primary school children in Kenya	• Water available schools: pupils showed no difference in period prevalence or duration of diarrhoeal illness<br>• Water scarce schools: pupils showed reduction in diarrhoea incidence and days of illness<br>• Mixed results on the impact of WASH in schools on diarrhoea experienced by pupils
[100]	Treating water with chlorine at point-of-use to improve water quality and reduce child diarrhoea in developing countries: a systematic review and meta-analysis	The American Journal of Tropical Medicine and Hygiene	February 2007	C	Level 2A: Systematic review of randomized controlled trials measuring the impacts of point-of-use chlorine water treatment on diarrhoea	• Reduced risk of child diarrhoea (OR 0.71)<br>• Reduced risk of stored water contamination with E. coli (OR 0.2)<br>• Majority of trials are relatively short so future studies with multi-year follow up are required to assess long term acceptability and sustainability of impacts
[101]	Use of ceramic water filtration in the prevention of diarrheal disease: a randomized	The American Journal of Tropical	November 2008	C	Level 1B: Randomized controlled trial determining the effectiveness of ceramic water filters in reducing diarrhoea	• Intervention households had significantly less detectable E. coli in

	controlled trial in rural South Africa and Zimbabwe	Medicine and Hygiene				their drinking water, with some having zero (57%)<br>• There was a lower incidence of diarrhoea in those using the water filters<br>• Results suggest ceramic filters are effective in reducing incidence of diarrhoeal disease
[102]	Water and hygiene interventions to reduce diarrhoea in rural Afghanistan: a randomized controlled study	Journal of Water and Health	December 2010	C, D, H	Level 1B: Randomized controlled trial of four interventions to create an evidence-base for water policy in Afghanistan	• Combination intervention group experienced a reduction in diarrhoea as compared to the control group<br>• Reduction was significant when all members of household were considered, but was not significant with only U5C<br>• Higher socioeconomic status was associated with reduced rate of diarrhoea and increased intervention uptake<br>• Multi-barrier methods are necessary due to opportunities for water contamination
[103]	Water filtration provision and home-based filter reinforcement reduce diarrhoea in Kenyan HIV-infected adults and their household members	The American Journal of Tropical Medicine and Hygiene	August 2014	C, H	Level 2B: Cohort study evaluating the effectiveness of water filters to reduce diarrhoea in HIV-infected adults	• Reduction in diarrhoea from 17.2% to 8.7% in participants 3 months post-intervention<br>• Reduction was similar to those taking a prophylaxis before being given a filter<br>• Implementing water filters in HIV care and treatment programmes may offer prophylactic benefit
[104]	Water, sanitation and hygiene for the prevention of diarrhoea	International Journal of Epidemiology	April 2010	A, C, E, F	Level 1A: Systematic review of randomized and quasi-randomized controlled trials evaluating the effect of various interventions on diarrhoea	• Effect of handwashing with soap is consistent across various study designs and pathogens but depends on access<br>(48% reduction risk)

						• Effect of water treatment appears large but not found in few blinded studies<br>(17% reduction risk)<br>• Rigorous evidence for benefit of sanitation (quasi-randomized) with 36% reduction risk
[105]	Water, sanitation, and hygiene interventions to reduce diarrhoea in less developed countries: A systematic review and meta-analysis	The Lancet Infectious Diseases	January 2005	A, C, E, H	Level 2A: Systematic review of papers studying any WASH interventions and diarrhoea morbidity as a health outcome in non-outbreak conditions	• All interventions significantly reduced risk of diarrhoea<br>• Most interventions had a similar impact on diarrhoea, with point-of-use water treatments found to be more effective than previously thought<br>• Multiple/combined interventions were not more effective than single interventions

Table S2. Coding for each type of intervention in Table S1

Level	Therapy/prevention, Aetiology/Harm
A	Engage in regular handwashing
B	Intake of prophylactic supplements
C	Engage in water treatment practice
D	Engage in water storage practice
E	Maintain household cleanliness
F	Manage household waste disposal appropriately
G	Community Infrastructure
H	Community Education and Participation
